# Dynamically Controlled Flight Altitudes in Robo-Pigeons via *Locus Coeruleus* Neurostimulation

**DOI:** 10.34133/research.0632

**Published:** 2025-03-05

**Authors:** Ke Fang, Zhouyi Wang, Yezhong Tang, Xiaofei Guo, Xing Li, Wenbo Wang, Bing Liu, Zhendong Dai

**Affiliations:** ^1^ Institute of Bio-inspired Structure and Surface Engineering, College of Mechanical and Electrical Engineering, Nanjing University of Aeronautics and Astronautics, Nanjing, China.; ^2^ Brainnetome Center and National Laboratory of Pattern Recognition, Institute of Automation, Chinese Academy of Sciences, Beijing, China.; ^3^ Chengdu Institute of Biology, Chinese Academy of Sciences, Sichuan, China.; ^4^ National Engineering Research Center for Nanomedicine, College of Life Science and Technology, Huazhong University of Science and Technology, Wuhan, China.

## Abstract

Robo-pigeons, a novel class of hybrid robotic systems developed using brain–computer interface technology, hold marked promise for search and rescue missions due to their superior load-bearing capacity and sustained flight performance. However, current research remains largely confined to laboratory environments, and precise control of their flight behavior, especially flight altitude regulation, in a large-scale spatial range outdoors continues to pose a challenge. Herein, we focus on overcoming this limitation by using electrical stimulation of the *locus coeruleus* (LoC) nucleus to regulate outdoor flight altitude. We investigated the effects of varying stimulation parameters, including stimulation frequency (SF), interstimulus interval (ISI), and stimulation cycles (SC), on the flight altitude of robo-pigeons. The findings indicate that SF functions as a pivotal switch controlling the ascending and descending flight modes of the robo-pigeons. Specifically, 60 Hz stimulation effectively induced an average ascending flight of 12.241 m with an 87.72% success rate, while 80 Hz resulted in an average descending flight of 15.655 m with a 90.52% success rate. SF below 40 Hz did not affect flight altitude change, whereas over 100 Hz caused unstable flights. The number of SC was directly correlated with the magnitude of altitude change, enabling quantitative control of flight behavior. Importantly, electrical stimulation of the LoC nucleus had no significant effects on flight direction. This study is the first to establish that targeted variation of electrical stimulation parameters within the LoC nucleus can achieve precise altitude control in robo-pigeons, providing new insights for advancing the control of flight animal–robot systems in real-world applications.

## Introduction

Pigeons exhibit exceptional maneuverability and adaptability through a distinctive combination of wing flapping and gliding, enabling flexible flight in diverse environments [1–3]. Inspired by the superior flight capabilities of pigeons, researchers have translated these biological insights into the design of aerospace vehicles, resulting in bioinspired flappingwing aircraft and microaerial vehicles (MAVs) [4–7]. However, existing MAVs still exhibit significant disparities compared to pigeons in terms of flight speed, environmental perception, energy supply, adaptability, and effective flight distance, thereby limiting their application in real-world mission scenarios. To overcome these limitations, in recent efforts by integrating microimplantable brain–machine interface technology with microelectromechanical systems (MEMSs), researchers have innovatively utilized pigeons as carriers to construct a bio-hybrid system known as the “robo-pigeon” [8–11]. This bio-hybrid system amalgamates the sensory, locomotor, and autonomous intelligence of the biological carrier with the high precision, repeatability, and controllability of MEMS [12–14], offering extensive prospects for the development of flexible and efficient aerial platforms.

Existing studies have successfully controlled various locomotor behaviors (such as turning, takeoff, and forward movement) in robo-pigeons under laboratory conditions through electrical stimulation of specific midbrain nuclei [15–18]. However, precise and reliable control of flight behavior in practical outdoor environments remains a critical challenge. In particular, controlling flight altitude continues to be an unresolved issue. Control of flight altitude not only directly impacts the robo-pigeon’s navigational capabilities within complex 3-dimensional spaces but also is closely linked to its adaptability, stability, and task execution efficiency in dynamic environments. For instance, when performing complex search and rescue tasks, the ability to flexibly adjust flight trajectories at different altitudes enables rapid responses to sudden obstacles or environmental changes. Thus, achieving precise altitude control is one of the core technologies needed to advance the application of robo-pigeons in real-world scenarios. However, current research on this issue is limited. Most studies have focused on controlling planar movements, and few have thoroughly investigated how to achieve precise altitude control in outdoor 3-dimensional environments.

Existing research has demonstrated that pigeons’ agile flight capabilities are associated with the intricate structures of their wing skeletons and muscles and are closely linked to the neural regulatory mechanisms within their brains [19–21]. During flight, pigeons dynamically adjust the frequency and amplitude of wing flapping to modulate aerodynamics, achieving a fine-tuned coupling of lift and thrust, thereby flexibly altering flight altitude and maintaining flight stability [22–24]. For example, increasing the flapping frequency and amplitude allows pigeons to generate greater lift to facilitate flight ascent, while decreasing these parameters can reduce lift production to maintain a stable flight descent [25–27]. This altitude control ability necessitates precise instructions and coordination of flight actions by the brain. Recent studies have identified that multiple nuclei within the midbrain play pivotal roles in regulating different flight behaviors in pigeons. For instance, electrical stimulation of *tractus septo-mesencephalicus* (TSM) and *archistriatum ventral* (AV) can effectively induce takeoff behavior [16], whereas stimulation of *formatio reticularis medialis mesencephali* (FRM) can precisely control the pigeon’s left and right turns [9,10]. However, these studies predominantly focus on planar motion control, lacking in-depth exploration of altitude control within complex 3-dimensional spaces.

Notably, our previous studies have shown that electrical stimulation of the *locus coeruleus* (LoC) in the pigeon’s midbrain can induce takeoff behavior in pigeons under awake conditions and regulate the rhythm of wing flapping movements under light anesthesia [15]. This suggests that the LoC nucleus can directly influence wing motion patterns and might regulate lift variations by adjusting wingbeat frequency and amplitude at the neural level. These finding provides critical insights into the neural regulatory mechanisms governing flight altitude. Existing studies have shown that norepinephrine (NE) released by the LoC nucleus participates in motor control and behavioral regulation across various vertebrates [28–30]. In mammalian models, LoC neurons interact with motor neurons and related central pattern generators (CPGs) in the spinal cord and brainstem through widely distributed NE projection fibers, thereby affecting muscle tone, motor rhythm, and motor coordination [31,32]. These CPGs are responsible for generating basic rhythmic actions such as gait, breathing, and swallowing, allowing NE modulation to alter overall motor behavior characteristics by adjusting their activity levels. Further research has also demonstrated that the LoC’s regulation of higher motor control centers, such as the thalamus, basal ganglia, and cerebellum, facilitates precise motor adjustments and strategy shifts in multi-task environments [33,34]. Although studies on the relationship between the LoC and motor control in birds are relatively limited, given the general influence of the LoC on motor behavior patterns in mammals [28], it is inferred that the LoC can similarly regulate flight-related neural circuits in birds, directly or indirectly affecting flapping frequency and amplitude, thereby achieving fine control of flight dynamics. Therefore, we have reason to hypothesize that electrical stimulation of the LoC nucleus can effectively control the flight altitude of robo-pigeons in outdoor environments, providing a novel research direction to address the challenges of 3-dimensional flight control.

**Fig. 1.**
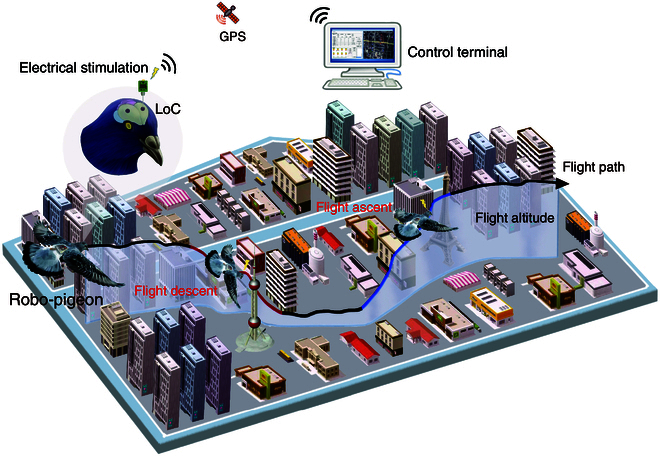
Schematic representation of the dynamic control of the flight altitude of a robo-pigeon by neural stimulation. In this study, the pigeon’s *locus coeruleus* (LoC) was used as the target nucleus for electrical stimulation, and by applying specific stimulation parameters, precise control of the ascending and descending flight altitude of the robo-pigeon could be realized.

To validate this hypothesis, the present study targets the LoC nucleus as the stimulation region, employing a multivariate repeated-measures analysis of variance (ANOVA) design to systematically investigate the effects of different stimulation parameters on the flight altitude control of robo-pigeons while also assessing safety and stability. The objective of this study is to achieve stable control of ascent and descent in robo-pigeon flight within actual outdoor scenarios (Fig. 1). We expect that this study will enhance the understanding of pigeons’ flight behaviors in complex environments, thereby providing a significant theoretical foundation and practical guidance for the development of flight control technologies in flying animal robots. Additionally, the findings are anticipated to have positive applications in fields such as search and rescue, environmental surveying, and scientific research [35,36].

## Results

### Stimulation in nucleus LoC and corresponding flight behaviors

**Fig. 2.**
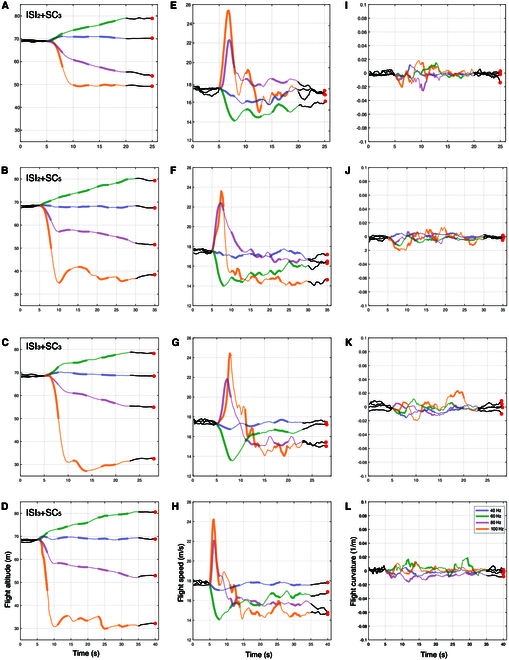
Dynamic changes in the flight characteristics of robo-pigeons under different stimulation parameters. (A), (E), and (I) depict the mean flight altitude, mean flight speed, and mean flight curvature dynamics over time, respectively, of the robo-pigeon under ISI_2_ + SC_3_ stimulation. (B), (F), and (J) show the mean flight altitude, mean flight speed, and mean flight curvature dynamics over time, respectively, of the robo-pigeon under ISI_2_ + SC_5_ stimulation. (C), (G), and (K) represent the mean flight altitude, mean flight speed, and mean flight curvature dynamics over time, respectively, of the robo-pigeon under ISI_3_ + SC_3_ stimulation. (D), (H), and (L) represent the mean flight altitude, mean flight speed, and mean flight curvature dynamics over time, respectively, of the robo-pigeon under ISI_3_ + SC_5_ stimulation. Among them, ISI denotes the interstimulus interval and SC denotes the stimulus cycles, with subscripts indicating the interval duration in seconds and the number of stimulus cycles, respectively. The black curve represents the 5 s before stimulation, and the black curve with red dots represents the 5 s following stimulation. Curves of different colors represent different SFs, with thick segments indicating stimulation periods and thin segments representing ISIs.

**Fig. 3.**
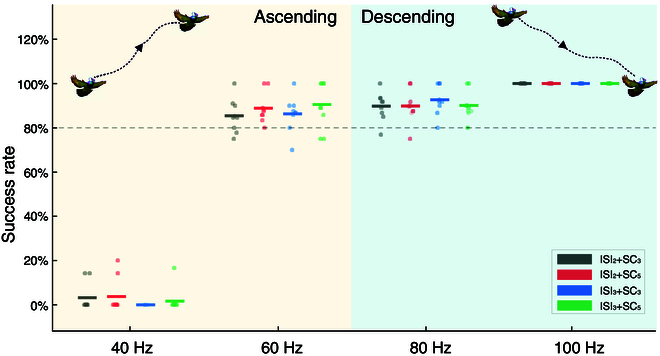
Average success rate of flight altitude control in the robo-pigeon under different stimulation parameters.

In all flight control experiments involving 8 robo-pigeons, our results indicated that changing the electrical stimulation parameters within the LoC nucleus effectively controlled the flight altitude of the robo-pigeons in the air. These changes in altitude were closely associated with alterations in flight speed but exhibited no significant impact on flight direction (Fig. 2 and Figs. S1 and S2). Specifically, the robo-pigeons could not actively control flight altitude ascent when the SF was set to 40 Hz for the LoC nucleus, irrespective of adjustments to SC or ISI parameters (Fig. 2A to D and Fig. S3). The average altitude increase was 1.065 ± 1.148 m, with a control success rate of only 2.20 ± 4.61% (Fig. 3 and Table S1). The flight trajectory of the robo-pigeons essentially remained straightly in the pre-stimulus flight direction (Fig. 2I to L and Fig. S3), with the flight speed insignificantly decreased (Fig. 2E to H and Fig. S3). Importantly, when the SF was set to 60 Hz, the robo-pigeons showed effective altitude ascent flight across all SC and ISI parameter settings (Fig. 2A to D and Fig. S4), with an average ascent altitude of 12.241 ± 1.761 m and a success rate of 87.72 ± 2.00% (Fig. 3 and Table S1). Its flight trajectory was relatively stable during these stimuli, with no changes in flight direction (Fig. 2I to L and Fig. S4), while the mean flight speed was significantly increased (Fig. 2E to H and Fig. S4).

Conversely, as long as the SF parameter was set to 80 Hz in the LoC nucleus, the descent of the robo-pigeons can be successfully controlled (Fig. 2A to D), with a gradual decrease in flight altitude and no significant change in flight trajectory direction (Fig. 2I to L and Fig. S5). Among them, the flight speed was first accelerated instantaneously and then gradually slowed down (Fig. 2E to H). During this stimulation condition, the average descent altitude was as high as 15.655 ± 2.516 m, with a high success rate of 90.52 ± 1.16% (Fig. 3 and Table S1). Notably, the similar control effects were observed with the 100 Hz stimulus (Fig. 2A to D), i.e., 100% success rate of control decreases in flight altitude (Fig. 3 and Table S1), but no obvious directional changes in the flight trajectory (Fig. 2I to L and Fig. S6). Compared to the 80 Hz stimulus, the descent flight of the robo-pigeon was much faster under this stimulation, achieving rapid 30 to 50 m descent within the first stimulus cycle (Fig. 2E to H and Fig. S6), which appeared as uncontrolled falling behaviors.

### Effects of stimulus parameters on flight altitude

**Fig. 4.**
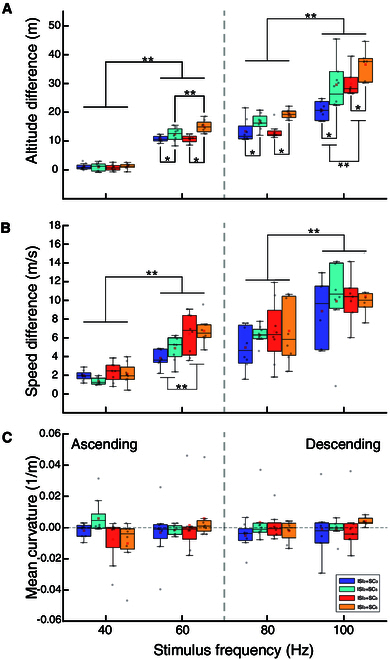
Quantitative comparison of flight variables in robo-pigeons under different stimulus parameters. (A) Average flight altitude difference. (B) Average flight speed difference. (C) Average flight curvature. Asterisks denote significant and highly significant differences (*P < 0.05 and **P < 0.001).

In this study, we analyzed the effects of stimulus parameters (SF, ISI, and SC) on changes of the flight altitude, flight speed, and flight curvature using a 3-factor repeated-measures ANOVA on 8 robo-pigeons (Fig. 4).

In the context of controlling the robo-pigeon’s flight altitude ascent, the SF and SC showed significantly main effects, whereas the ISI parameter did not have a significant main effect (Table S2). There were significant interaction effects between SF and SC, and between ISI and SC (Table S3). No significant interaction effects between SF and ISI, and among SF, ISI, and SC were found (Fig. 4A). Simple–simple effects analyses showed that in 2 SC conditions the increased degree of flight altitude was significantly greater with 60 Hz stimulation than those with 40 Hz, while in 2 ISI conditions SC_5_ could drive the robo-pigeons to climb higher than SC_3_ did (Fig. 4A and Table S3).

For the control of flight altitude descent, significant main effects were observed for SF, ISI, and SC parameters (Fig. 4A and Table S2). There was a significant interaction effect between SF and ISI, but no effect among SF, ISI, and SC (Fig. 4A and Table S3). The multiple comparisons further showed that 100 Hz stimulation was significantly more effective than 80 Hz in controlling flight altitude descending, and that the amplitude of flight altitude descending was correlated with an increase in the SC and ISI parameters (Fig. 4A and Table S3). Increasing ISI could lead to the greater decrease in flight altitude for the robo-pigeons with 100 Hz stimulation, but no such effect was found with 80 Hz (Fig. 4A and Table S3). Our results suggested that SF played a pivot role in initiating and maintaining either ascending or descending flight, while SC is the main factor to manipulate the change magnitude of flight altitude. In general, flight speed was closely related to changes in flight altitude. SF and ISI showed significant effects during flight ascent control, with 60 Hz stimulation producing faster speeds than 40 Hz, and further acceleration occurring with larger ISI and SC values (Fig. 4B and Table S2). During flight descent control, only SF had a significant effect, with 100 Hz inducing greater speed than 80 Hz (Fig. 4B and Table S2). No significant effects of the stimulus parameters (SF, ISI, and SC) on flight curvature were observed during either ascent or descent (Fig. 4B and Table S2).

## Discussion

This study demonstrates that different neural stimulation parameters (SF, SC, and ISI) applied to the LoC of pigeons in outdoor environments can effectively modulate their flight altitude. These findings provide new evidence for the regulatory mechanisms of the LoC nucleus in vertebrate central nervous system (CNS) motor control. As a major NE center in the brain and a key component of the reticular activating system, the LoC nucleus plays a crucial role in the CNS [28,30,34,37]. It innervates the spinal cord, brainstem, cerebellum, hypothalamus, relay nuclei of the thalamus, amygdala, basal part of the telencephalon, and the cerebral cortex, and plays crucial roles in arousal, attention, stress response, and motor control [30,38]. Previous studies on motor neurons in cats’ hindlimbs revealed a specific functional relationship between LoC nucleus and spinal motor neurons, establishing LoC nucleus’ central role in the spinal motor pathway [34,39,40]. Although these studies have focused primarily on mammals, many of the fundamental properties and functions of the LoC nucleus exhibit a high degree of conservation in other vertebrate models (fish, amphibians, reptiles, and birds) [28,41–44].

Previous studies on mildly anesthetized pigeons found that microstimulation of the LoC nucleus could modulate wing flapping. In awake pigeons, electrical stimulation of the LoC nucleus induced take-off behavior in an indoor, ground-level environment. In contrast, the present study, conducted in an outdoor flight setting, showed that LoC nucleus stimulation can induce both ascending and descending flight behaviors. This striking difference highlights the substantial variation in motor control strategies pigeons employ in response to differing environmental conditions. It suggests that pigeons exhibit heightened environmental sensitivity and accordingly select distinct neuromodulatory mechanisms and motor response patterns. In terrestrial locomotion, animals must navigate complex terrains, obstacles, and environmental variables, which may selectively activate LoC subregions involved in coordination and balance [34,45,46]. Conversely, in the more dynamic, multidimensional, and constantly changing outdoor flight environment, pigeons rely on LoC subregions associated with flight maneuverability and spatial perception. Compared to a relatively stable indoor environment, outdoor conditions demand more flexible adjustment of flight states and navigation capabilities, resulting in priority activation of neural pathways that meet such complex flight requirements. Thus, the differences in behavioral responses induced by varying SFs reflect the robo-pigeons’ adaptive flight control strategies under distinct environmental contexts. Specifically, 60 Hz stimulation effectively activates neural circuits associated with ascending flight, whereas 80 Hz stimulation triggers circuits related to descending flight. This environment-dependent neural activation pattern closely parallels the LoC mechanism of selectively activating particular brain regions under different behavioral states. Different SFs induce distinct neural networks, resulting in frequency-dependent behavioral mode shifts. For example, recent optogenetic stimulation of the LoC nucleus studies in mice have shown that a 3 Hz tonic firing mode primarily activates the prefrontal cortex, associated with cognitive flexibility, whereas 5 Hz firing more strongly activates the amygdala and thalamus, modulating emotional and stress responses [47]. Such frequency-dependent, nonlinear activation patterns support the role of the LoC nucleus in regulating diverse behaviors. Notably, at 40 Hz stimulation, the robo-pigeon showed no obvious response, possibly because this frequency did not sufficiently excite LoC-related neurons and circuits. However, when the SF exceeded 100 Hz, a precipitous descent was induced, likely due to LoC neuronal overactivation. Such overactivation may cause neuronal fatigue, reduce neuronal responsiveness, and inhibit circuits responsible for maintaining balance, leading pigeons to perceive a loss of equilibrium and trigger an emergency descent. This response is analogous to the rapid descent observed when birds encounter threats or lose balance in natural environments [48].

Generally, avian flight altitude control generally depends on precise flight posture adjustments, which essentially involve highly complex aerodynamic and physiological coordination mechanisms [27,49,50]. During ascent, birds typically increase wingbeat amplitude and frequency to achieve greater lift and higher flight speed, thereby overcoming gravity and attaining higher altitudes. In contrast, during descent, birds reduce wingbeat amplitude and frequency, resulting in lower lift and slower flight speed, allowing for a gradual decrease in altitude [23,51–53]. In this study, we found that the same aerodynamic principles apply to robo-pigeons: During ascent, flight speed is positively correlated with climbing altitude, whereas during descent, flight speed decreases as altitude is reduced. The SF parameter acts as a “switch” within this process, as different SFs induce distinct altitude control modes and dynamic flight characteristics. As with the LoC’s selective modulation of specific brain regions through particular firing patterns, this frequency-dependent “switch” effect is closely related to the neuromodulatory functions of the LoC nucleus. As the brain’s primary noradrenergic (NE) center, the LoC nucleus plays a critical role in sensory processing and motor production, and its principal neuronal activity modes can be broadly divided into tonic and phasic [31,37,40]. Tonic activity helps maintain vigilance and attention, fundamental for basic motor skills and bodily stability. In contrast, phasic activity, characterized by brief bursts in response to specific stimuli or events, enables rapid responses to new environmental information. Our results suggest that different SFs may selectively activate the phasic firing mode of LoC neurons, thereby inducing distinct flight state transitions. Such frequency-selective neuromodulatory mechanisms have been corroborated by studies in other animal models and brain regions. For example, electrical stimulation studies in the rat hippocampal CA1 region have shown that low-frequency (10 to 20 Hz) stimulation tends to reduce principal neuron firing rates, whereas high-frequency (100 to 400 Hz) stimulation increases both principal and interneuron firing, leading to markedly different physiological and behavioral outcomes [54]. Similarly, in the pig mesencephalic locomotor region (MLR), 20 Hz low-frequency stimulation induces exploratory behavior, whereas 50 Hz high-frequency stimulation elicits more intense and rapid running behaviors [55]. These findings align with a broader body of neuromodulation research, indicating that SF can alter the dynamic properties of neural circuits [32,56].

By contrast, the SC parameter plays a pivotal role in quantitatively regulating the amplitude of altitude changes during ascent and descent in the robo-pigeon. The interaction between SF and SC likely reflects a combination of frequency-driven fundamental patterns and incremental effects regulated by SCs. While SF primarily influences the direction of flight responses by modulating neuronal firing patterns, SC fine-tunes the amplitude of flight movements by adjusting the duration and intervals of electrical stimulation. For example, when keeping the frequency constant and varying the SC, we can regulate the robo-pigeon’s flight altitude, resulting in greater or smaller altitude changes in the same direction. This suggests that SC parameters act as an “amplitude modulator” of movement amplitude, enabling more precise control of motor actions through sustained stimulation of the nervous system. The regulation of flight altitude by SC may be linked to the integrative mechanisms of LoC neurons. Previous studies have demonstrated that LoC neurons are capable of integrating input signals across different time scales to produce corresponding output patterns [29,31,34]. In the context of flight movements, shorter SCs may not provide sufficient time for excitatory accumulation in neurons, resulting in smaller movement amplitudes. In contrast, longer SCs may allow excitatory signals to accumulate adequately, leading to larger motor responses. This mechanism is consistent with our observations in the robo-pigeon flight experiments and further highlights SC parameters as a key factor in the modulation of motor behavior.

Although ISI parameters had a limited effect on altitude regulation in this study, a result that may be attributed to the narrow parameter ranges tested, increasing ISI could still prove advantageous from a neuromodulatory standpoint. Adjusting ISI may alleviate neuronal fatigue caused by continuous electrical stimulation, thereby enhancing the sustainability and stability of stimulation over time. Prolonged stimulation may lead to neuronal fatigue, manifested as a reduced response to stimuli, which can compromise the stability of motor behavior [48,57]. Appropriately setting ISI to allow sufficient recovery time between stimuli can alleviate fatigue and maintain normal firing patterns in neurons. For instance, LoC neurons are prone to fatigue under high-frequency stimulation, and insufficient ISI may weaken their regulatory capacity. Studies have shown that increasing ISI can extend the neuron’s recovery time, allowing it to maintain effective firing patterns during subsequent stimulations, thus enhancing the sustainability of the stimulation and preventing abnormal behaviors resulting from neuronal fatigue [58,59]. In this study, a moderate increase in ISI could provide the LoC neurons with additional recovery time, thereby reducing fatigue during prolonged stimulation and maintaining the stability and sustainability of flight behavior. This is particularly important in long-term or high-frequency electrical stimulation applications, especially in persistent tasks or behavioral modulation, where it is critical to ensure that neurons are able to maintain their functional responses under continuous stimulation [59–61]. Future research could further explore how precise modulation of ISI affects fatigue and behavior in different neuronal populations, providing theoretical insights for optimizing neurostimulation protocols.

From a practical applications perspective, precise control of robo-pigeon flight altitude is essential for stable operation in variable outdoor environments. Based on our findings, optimizing stimulation parameters is crucial to ensuring accuracy, stability, and safety in flight altitude control. Safety is the primary consideration in the outdoor flight of robo-pigeons. To ensure effective altitude control and avoid excessive neural stimulation, it is advisable to set a safe SF range of 40 to 100 Hz. Within this range, specific frequencies can be pre-selected according to mission requirements, allowing control over ascending or descending flight modes. To further refine flight control accuracy, SC parameters can be adjusted. Compared to short-cycle stimulation (SC_3_), long-cycle stimulation (SC_5_) can induce greater altitude changes. By increasing or decreasing SC, users can precisely control altitude amplitude, adapting the robo-pigeon to various flight scenarios. For tasks requiring prolonged altitude stability, shorter SCs can maintain stable flight, whereas longer cycles suit rapid altitude adjustments. For example, when the robo-pigeon must cruise at a fixed altitude, shorter cycles combined with moderate frequency settings can minimize altitude fluctuations, thereby enhancing flight stability.

Additionally, our study revealed a strong correlation between flight altitude and flight speed, emphasizing the need for integrated control strategies. This is particularly important during rapid descents to ensure safety. The study found that 80 Hz stimulation effectively induces a slow descent, whereas high-frequency stimulation at 100 Hz may lead to an excessive increase in speed, posing a risk of uncontrolled descent. To avoid such risks, a lower frequency (80 Hz) should be prioritized, coupled with extended SCs to achieve smooth altitude reduction and safe landing. This synergistic control strategy of altitude and speed is particularly critical in scenarios such as environmental monitoring and search and rescue missions. Especially when the robo-pigeon is in situations requiring prolonged low-speed flight, precise control of flight speed ensures the successful completion of tasks. Although the ISI parameters do not directly affect altitude regulation, they are critical in maintaining flight stability in robo-pigeon movement control. Appropriately extending ISI can prevent overstimulation of LoC neurons, thus preserving the stability of neural responses during prolonged flight tasks. Especially, in long-duration missions, such as aerial patrol or search and rescue, increasing ISI can effectively prolong flight control endurance and reduce the negative impacts of neural fatigue on flight behavior. In short-duration high-intensity missions, such as emergency altitude adjustment or rapid response, ISI can be appropriately shortened to ensure the sensitivity and efficiency of the system response.

However, the sample size used in this study is relatively small. Although our results are statistically robust and reliable, this limitation restricts the generalizability and comprehensiveness of our understanding of LoC-mediated neural regulation mechanisms. Furthermore, while this study provides behavioral-level evidence of the impact of LoC stimulation parameters on flight altitude, the current lack of direct neurophysiological recording data further hinders our precise understanding of the underlying LoC mechanisms involved in flight control. Therefore, future research should aim to expand the sample size and incorporate a broader range of experimental conditions while also considering the integration of electrophysiological recording techniques into outdoor experiments. This will elucidate the specific mechanisms by which stimulation parameters regulate neuronal excitability, thereby providing a more robust neurobiological foundation for optimizing stimulation protocols and ultimately enhancing the precision and safety of flight control.

Furthermore, environmental factors such as wind speed, temperature, and visibility may influence the outcomes of flight altitude control experiments. Although this study conducted experiments under relatively standardized conditions and standardized flight data to reduce the interference of environmental and individual differences, the potential impact of environmental factors still warrants further investigation. Future research should incorporate these environmental variables as covariates in the statistical models to more accurately assess the independent effects of stimulation parameters on flight altitude. Meanwhile, in practical application scenarios, integrating real-time environmental sensors (e.g., anemometers and thermometers) with adaptive control algorithms to dynamically adjust stimulation parameters in response to environmental fluctuations will further enhance the robustness and reliability of robo-pigeon flight control under complex conditions.

In summary, this study provides compelling evidence that LoC electrical stimulation can effectively regulate the flight altitude of robo-pigeons and elucidates the potential mechanisms by which stimulation parameters (SF, SC, ISI) influence flight altitude control. These findings lay a crucial foundation for advancing neural regulation technologies in applications such as flight navigation, search and rescue, and other critical operations.

## Materials and Methods

### Animals

All homing pigeons (*Columba livia*) were housed in the laboratory’s rooftop loft and were subjected to normal day and night light conditions. Water, gravel, and standard pigeon food mixes were well supplied and freely available to the pigeons. To train the pigeons to adapt to the weights, a dummy weight nut was attached to their backs using Velcro straps, which weighed 16 g (almost the same size and weight as the on-board control module). Every morning and evening, they were trained with 2 flights around the loft. Before the experiment started, 15 homing pigeons of unknown sex (aged 1 to 1.5 years) were selected for long-distance homing training for at least 2 weeks to ensure that all subjects had homing experience. All homing pigeons were transported for release training to Fangshan, Nanjing (118.78784°E, 31.93707°N), which is about 11.6 km from the loft. At the end of the training phase, we selected those pigeons with a stable homing path as candidates for the robo-pigeon based on the flight path data of these homing pigeons.

### Surgery

**Fig. 5.**
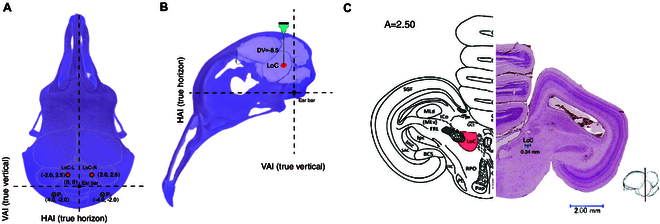
Electrode implantation sites in the pigeon brain. (A) Implantation position of the electrode on the surface of the pigeon skull. (B) Implantation depth of the electrode into the LoC nuclei within the pigeon brain. (C) Position of the electrode tip in a coronal section of the pigeon brain. LoC-L and LoC-R represent the LoC (*locus coeruleus*) nucleus in the left and right hemispheres, respectively. P1 and P2 indicate the positions of miniature stainless steel screws implanted above the lateral aspects of the biparietal suture.

Based on the above results of flight training in homing pigeons, a total of 8 pigeons (weight 435 ± 35 g) with stable homing experience were selected for brain electrode implantation in this study. The pigeons were fasted for 24 h before the start of the surgery, during which time they were guaranteed adequate water intake. All surgical procedures used in this study are described in detail in our previous studies [10,15]. Briefly, the combined anesthesia method was used for the brain surgery experiment, in which the subject pigeons were injected with 1.5% sodium pentobarbital solution (1.5 ml/500 g body weight) at the pectoral muscle for general anesthesia, and 2% lidocaine hydrochloride solution (0.5 ml) was injected subcutaneously in the surgical area of the brain for local anesthesia. The degree of anesthesia was evaluated by the toe-clamping response during the entire experiment. After the pigeons were fully anesthetized, their heads were fixed in a specially designed brain stereotaxic apparatus (type 68027, RWD Life Science, Shenzhen, China) with the anterior fixation point (i.e., rostral bar position) located 45° below the horizontal axis of the apparatus. In this study, the LoC nucleus was selected as the only microstimulation target, and the spatial coordinate position of the LoC was determined according to the pigeon brain atlas (AP: 2.5 mm, ML: 2.5 mm, DP: 8.5 mm) [62], where AP refers to the anterior–posterior position, ML denotes the medial–lateral position, and DP indicates the dorsal–ventral position, for implantation of stimulation electrodes (Fig. 5A and B). A 100 μm diameter polyformaldehyde insulated nickel–chromium alloy wire was selected as the stimulating electrode and implanted in the LoC nucleus areas of the right and left brains at one end, and the other end was fixed by tin soldering to the electrode adapter plate. All electrode implantation sites were sealed with α-cyanoacrylate fast medical adhesive (EC glue) to seal the gap between the electrode and the skull. Two screws were implanted at P_1_ and P_2_ on the skull surface (about 5 mm below the skull) for fixation of the electrode adapter plate, and a metallic silver wire was wound as the ground wire. After the electrode implantation was completed, the electrode adapter plate in the brain of the homing pigeon was fixed with dental acrylic resin. Each robo-pigeon was kept individually for 6 d for recovery after the procedure, and then further experiments were conducted.

At the end of all experiments, 4 subjects were randomly selected and euthanized by blue dot marking and injection of an overdose of pentobarbital sodium solution, followed by carotid artery perfusion to immobilize the brain with 75% saline and 4% formaldehyde solution. Subsequently, the brain was taken in brain stereotaxic positioning and subjected to histological analysis including sectioning and staining to confirm the correct positioning of the electrodes on the LoC nuclei (Fig. 5C) to eliminate unexpected data. All experimental procedures were approved by the Jiangsu Provincial Association of Laboratory Animal Science (license no.: 2010012, date: 2010), and all were performed following the Regulations of China’s Guide for the Management of Laboratory Animals to minimize animal suffering.

### Stimulation protocol

The stimulation parameters were determined based on previous laboratory trials and outdoor pre-experiments (Fig. S7 and Table S4). Indoor tests demonstrated that the robo-pigeons’ takeoff behavior was closely linked to the SF, whereas no significant correlation was observed with the stimulation duration (SD). Specifically, our indoor results revealed a marked threshold effect of SF on the initiation of flight: Below 40 Hz, no distinct flight responses were observed; at 60 Hz, the pigeons consistently assumed a “pre-takeoff” posture (characterized by slightly spread wings and a crouching stance); and at frequencies exceeding 80 Hz, they exhibited complete takeoff behaviors. Furthermore, outdoor pre-experiments confirmed that when SF surpassed 120 Hz, the risk of unintended flight behavior increased substantially, compromising the pigeons’ ability to return safely.

**Fig. 6.**
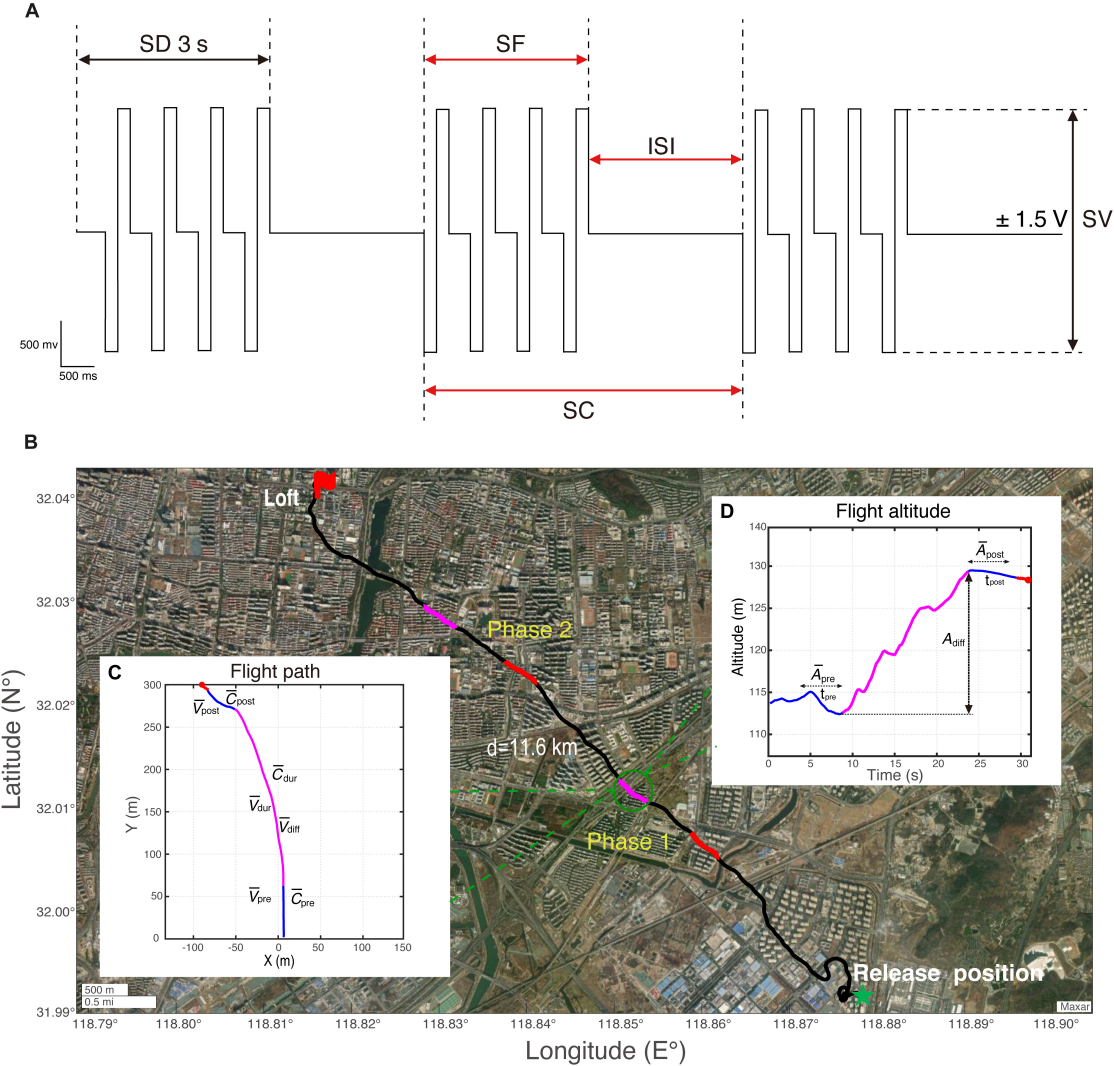
Experimental design for outdoor flight control of the robo-pigeon and quantitative analysis of its flight trajectory. (A) Design of the experimental stimulus parameters, where black arrows denote fixed parameters and red arrows denote adjustable stimulus parameters. (B) Outdoor flight altitude control experiment, with the black line indicating the robo-pigeon’s homing path, while the red and magenta lines correspond to the stimulus parameter control at different experimental phases. Insets (C) and (D) display the flight trajectory and altitude changes of the robo-pigeon under these stimulus conditions, along with the quantitative analysis of its flight data. *t*_pre_, first 5 s of the stimulus segment; *t*_post_, post-stimulus 5 s of the stimulus segment. $\bar{V}$_pre_, $\bar{C}$_pre_, and $\bar{A}$_pre_ denote mean flight speed, mean flight curvature, and mean flight altitude during the *t*_pre_, respectively. $\bar{V}$_dur_ and $\bar{C}$_dur_ denote mean flight speed and mean flight curvature during the stimulus, respectively. $\bar{V}$_diff_ and $\bar{A}$_diff_ denote the changes in flight speed and flight altitude before and after the stimulus, respectively. $\bar{V}$_post_, $\bar{C}$_post_, and $\bar{V}$_post_ represent mean flight speed, mean flight curvature, and mean flight altitude during *t*_post_, respectively.

To investigate the influence of LoC nucleus stimulation on outdoor flight altitude control under safe operating conditions, we identified 3 key parameters: SF, ISI, and SC (Table). The SF was set at 4 levels (40, 60, 80, and 100 Hz) to cover the progression from “no apparent flight response” (≤40 Hz) to “pre-takeoff” (60 Hz) and “reliable takeoff” (≥80 Hz), with 100 Hz serving as a higher boundary to avoid compromising flight safety. In addition, to mitigate the risk of neural tissue damage caused by excessive stimulation [59,63], we employed unit stimulation pulse trains in which ISI was assigned 2 levels (2 s, 3 s), and SC was set at 2 levels (3, 5). All other parameters remained constant: The stimulation waveform (SW) was a biphasic rectangular wave (negative–positive), the stimulation voltage (SV) was maintained at ±1.5 V, and SD was fixed at 3 s (Fig. 6A). This design ensures that altitude modulation in real-world outdoor environments can be thoroughly examined within a safe range, thereby enabling a controlled study of the effects of LoC nucleus stimulation on robo-pigeon flight behavior.

**Table.** Experimental levels of experimental parameters for controlling the flight altitude of robo-pigeons

SV	SW	SD	Stimulus variables	Unit	Test level*s*
±1.5 V	Negative–positive biphasic square pulse train	3 s	SF	Hz	40, 60, 80, 100
			ISI	s	2, 3
			SC	Num	3, 5

In addition, to minimize the influence of electrode implantation position deviation and interindividual differences on the experimental results, the initial parameters such as SF, SD, and ISI were fixed and controlled before each outdoor experiment. Meanwhile, a specific stimulation pulse width was set for each robo-pigeon according to the principle of “minimum effective dose” to ensure that the desired motor behavior could be triggered under the lowest effective stimulation intensity [64,65]. Under these conditions, the interference between neighboring electrodes can be minimized and tissue damage at the electrode interface can be avoided, thus improving the controllability and repeatability of the stimulation effect. It is worth noting that the stimulation pulse width required to achieve the same induced behavior shows an increasing trend as the number of experimental days and the frequency of testing increase. To ensure the comparability of results across experiments, the present study uniformly used a unitary stimulus sequence with equivalent effects in all experiments, thus ensuring that the initial takeoff behavior of each subject remained consistent within the same stimulus cycle (“pre-takeoff” state). The overall stimulus parameters were studied using a 3-way repeated-measures ANOVA experimental design.

### Experimental procedures

All outdoor flight altitude control experiments for the robo-pigeons were centrally conducted from September 2021 to June 2022. To ensure the consistency of environmental conditions, we selected clear weather with wind speeds below 2 m/s and conducted all experiments between 7:00 and 8:00 AM. Before each experiment, detailed records of weather conditions (including temperature, humidity, wind speed, and visibility) were collected to confirm that sunlight and weather variability did not adversely affect the robo-pigeons’ flight performance. By enforcing these stringent criteria, we aimed to minimize potential environmental fluctuations. The release experiment procedures for the robo-pigeon were similar to those described previously [10]. To enable the pigeons to return quickly, we wrapped the release cage with a black curtain during its movement from the loft to the release site to minimize the pigeons’ opportunity to observe the outside environment, which facilitated its quick return to the loft after release. Once at the release site, the pigeons were released after 15 min to minimize their hoovering behavior at the release site. Additionally, to ensure the acquisition of effective flight data, the parametric stimulation experiments were conducted only when the robo-pigeon’s flight trajectory approximated a straight line. This approach minimized confounding effect associated with autonomous navigation, thereby enhancing the interpretability of the experimental results.

The whole experimental procedure was divided into 2 phases (Fig. 6B), each with 2 stimulation tests. Between these 2 tests, stimulation was suspended for 60 s to allow the pigeons’ brain neural activity to return to a resting equilibrium state and to prevent fatigue caused by prolonged stimulation. Additionally, a 5 min interval rest period was set between the 2 experimental phases to ensure that the flight trajectories of the robo-pigeons tend to a straight line again before the next experiment, thus facilitating the subsequent flight trajectory control experiments. Given that sequential effects and pseudo-replication in animal behavioral and neuroscience studies may affect the robustness of statistical analyses [66,67], the present study employed a pseudo-randomized sequence for the order in which stimulus parameters were tested in each flight experiment. Therefore, this study employed a pseudo-randomized sequence for the order in which stimulus parameters were tested in each flight experiment. Furthermore, no less than 5 repetitions of each parameter level were performed to minimize the influence of the complex and variable outdoor environment on the experimental results and to ensure the validity and reliability of the statistical analysis results.

### Data acquisition and processing

To achieve precise control of the robo-pigeon’s outdoor flight altitude, this study employed a miniature neurostimulator measuring 23 mm × 20 mm × 5 mm and weighing approximately 6.3 g, based on a single-layer printed circuit board (PCB) structure (Fig. S8A and B). This device integrated a stimulation generator module, a GPS positioning module, and a barometric pressure sensor module, and has the functions of stimulus signal output, flight trajectory, flight altitude, and flight time data storage (Fig. S8C). Among them, the GPS module offered a positional accuracy of ±1 m, and the height measurement error of the barometric pressure sensor was within 1 m. During the experiments, the neurostimulator’s sampling rate was set at 5 Hz to record the robo-pigeons’ flight trajectories and altitudes data under various stimulation parameters.

The flight data were analyzed following the methodology described by Nagy et al. [68]. First, the robo-pigeons’ recorded flight trajectories from geographic coordinates (latitude and longitude) were converted into Universal Transverse Mercator (UTM) coordinates. The resulting raw flight trajectory and altitude data were then smoothed using a moving average filter (MAF) with the window size set to 3 points to strike a balance between effective noise reduction and preservation of short-term behavioral variations. Any missing data points were subsequently replaced using mean interpolation to maintain data continuity. After these preprocessing steps, changes in flight altitude during the stimulation period were assessed. Cases in which altitude variations remained consistently within ±5 m were considered indicative of autonomous flight adjustments rather than stimulus-induced responses, and were thus classified as invalid regulation events. In contrast, data displaying altitude deviations exceeding ±5 m during stimulation were identified as instances of successful control, suggesting that the applied stimulation effectively modulated the robo-pigeons’ flight altitude.

Then, all the recorded valid data were validated to calculate the success rate of the robo-pigeon's flight altitude control under different stimulus parameter conditions. Meanwhile, the flight data at 5 s before stimulation (*t*_pre_) and 5 s after stimulation (*t*_post_) were selected as positive controls, respectively. For quantitatively evaluating the effects of various stimulation parameters on the flight altitude and the dynamic changes of the flight characteristics of the robo-pigeon, we separated statistically and calculated the flight characteristics values of each subject based on the flight trajectories of the robo-pigeon, including the mean flight speed ($\bar{V}$_pre_), the mean flight curvature ($\bar{C}$_pre_), and the mean flight altitude ($\bar{A}$_pre_) in the 5 s before the stimulation; mean flight speed ($\bar{V}$_dur_), mean flight curvature ($\bar{C}$_dur_), mean flight speed difference ($\bar{V}$_diff_), and mean flight altitude difference ($\bar{A}$_diff_) during the stimulation period; and mean flight speed ($\bar{V}$_post_), mean flight curvature ($\bar{C}$_post_), and mean flight altitude ($\bar{A}$_post_) during the post-stimulus period (Fig. 6C and D), where the mean flight speed and mean flight curvature are analyzed with reference to the formulae calculated in previous studies.

Furthermore, considering the differences in flight capabilities and daily outdoor environments among individual robo-pigeons, these variations may lead to significant fluctuations in flight altitude across different experimental trials. To mitigate the impact of such variability on subsequent statistical analyses, we first conducted a comprehensive analysis of each subject’s flight behavior data recorded during the 5 s preceding stimulation. From this analysis, we extracted 3 key flight characteristics variables: total mean flight speed, total mean flight curvature, and total mean flight altitude. Subsequently, we used these flight characteristics as benchmarks to standardize the flight data of each robo-pigeon throughout the entire stimulation phase (including pre-stimulation, stimulation, and post-stimulation periods). This standardization effectively eliminates numerical deviations caused by individual differences in flight capabilities and reduces the influence of external environmental factors (such as wind speed and temperature) that may vary between different experimental days or flight batches. Importantly, this normalization process facilitates the quantitative comparison of flight data by aligning each robo-pigeon’s flight metrics to a common baseline, thereby providing a consistent measurement scale for subsequent variance analyses. All data analysis was conducted in Matlab 2022b (The MathWorks Inc., Natick, MA, USA).

### Statistical analyses

All subjects’ flight data were expressed as the mean ± SE. Prior to conducting the main statistical analyses, the Shapiro–Wilk *W* test and Levene’s test were used to test for distribution normality of means and test for homogeneity of variance for the $\bar{V}$_diff_,$\;\bar{C}$_dur_, and $\bar{A}$_diff_ of the 8 robo-pigeons, respectively. As these data satisfied the assumptions of normal distribution and homogeneity of variance, a 3-factor repeated-measures ANOVA was performed to further investigate the effects of different stimulation parameters. Specifically, “SF,” “ISI,” and “SC” served as the factors in the model, allowing for the evaluation of both main effects and interaction effects among these parameters. If the interaction was significant, further simple effects analysis or simple–simple effects analysis was performed. For post hoc comparisons, the least significant difference method was utilized. If Mauchly’s test indicated a violation of sphericity, the Greenhouse–Geisser correction was applied to adjust the degrees of freedom accordingly. Effect sizes were detected by partial η^2^ (0.20 for low effect sizes, 0.50 for medium effect sizes, and 0.80 for high effect sizes). In addition, we correlated the flight eigenvalues under different stimulus parameters using Pearson correlation analysis. Additionally, pearson correlation analyses were performed to explore the correlation among flight eigenvalues under different stimulation parameters. All statistical analyses were conducted using IBM SPSS Statistics 26.0 (IBM Corporation, Armonk, NY, USA), and *P* values were marked as statistically significant as follows: *P < 0.05 and **P < 0.001.

## Data Availability

All data are available in the main text or the Supplementary Materials, and can be available from the corresponding author upon reasonable request.
