# Supplementary material for: Dynamically Controlled Flight Altitudes in Robo-Pigeons via Locus Coeruleus Neurostimulation
Source: Research (Wash D C). 2025 Mar 5;8:0632. doi: 10.34133/research.0632 (PMC11880575; doi:10.34133/research.0632)
Supplement: Supplementary 1 — Figs. S1 to S8 Tables S1 to S4 [file research.0632.f1.zip › 02 Supplementary Materials.docx]

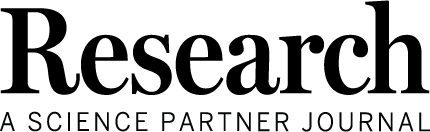


**Supplementary Materials**

**Dynamic Controlled Flight Altitudes in Robo-Pigeons via Locus Coeruleus Neurostimulation**

Ke Fang^1, 2^†, Zhouyi Wang^1^†*, Yezhong Tang^1, 3^, Xiaofei Guo^1^, Xing Li^2, 4^, Wenbo Wang^1^, Bing Liu^2^* and Zhendong Dai^1^

^1^ Institute of Bio-inspired Structure and Surface Engineering, College of Mechanical and Electrical Engineering, Nanjing University of Aeronautics and Astronautics, China.

^2^ Brainnetome Center and National Laboratory of Pattern Recognition, Institute of Automation, Chinese Academy of Sciences, China.

^3^ Chengdu Institute of Biology, Chinese Academy of Sciences, China.

^4^ National Engineering Research Center for Nanomedicine, College of Life Science and Technology, Huazhong University of Science and Technology, China.

^*^Address correspondence to: Bing Liu; bing.liu@ia.ac.cn and Zhouyi Wang; wzyxml@nuaa.edu.cn

† These authors contributed equally to this study

**This PDF file includes:**

Supplementary Text

Figs. S1 to S8

Tables S1 to S4


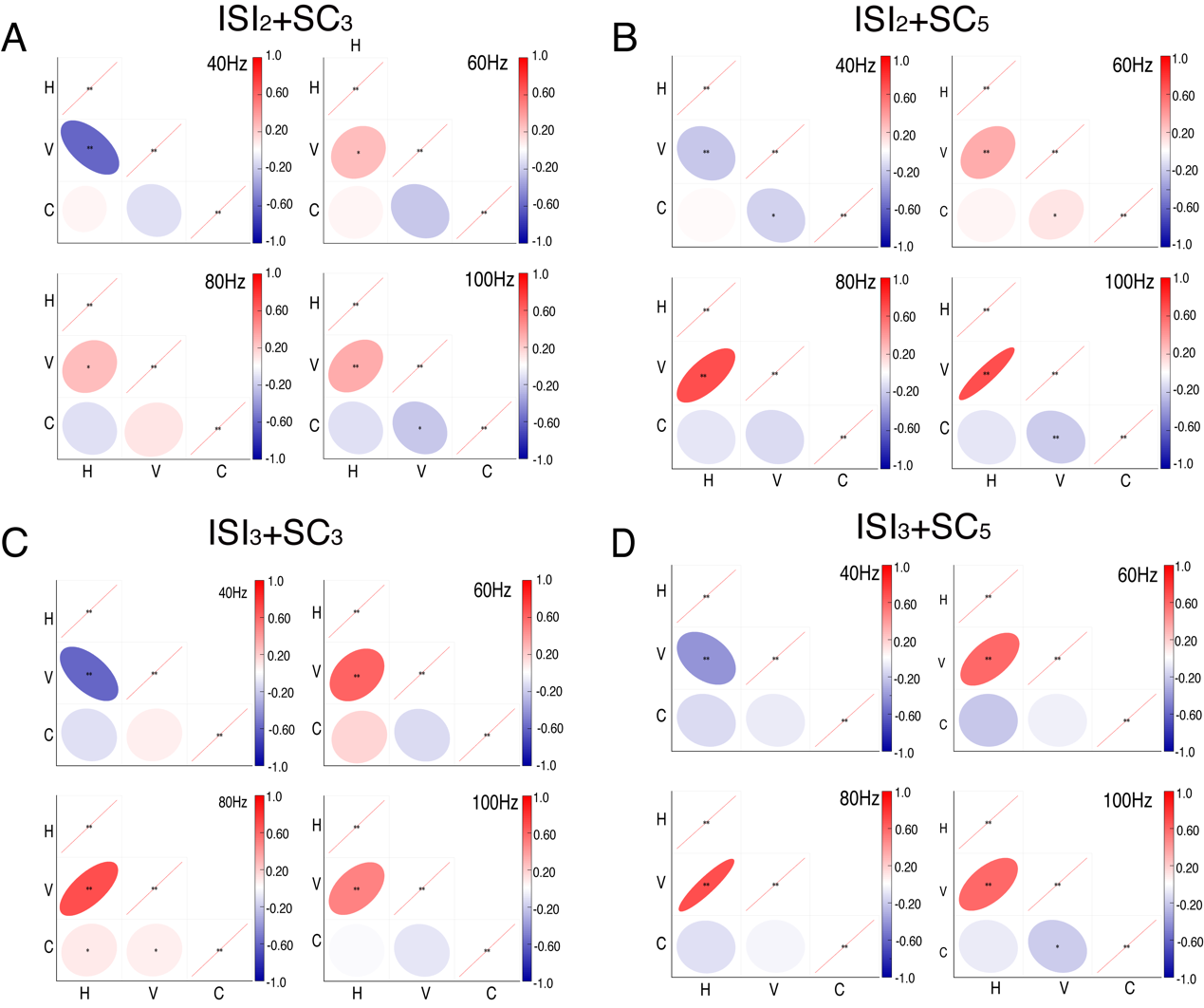
**Fig. S1.** Heatmap of correlations between flight variables of the robo-pigeon under different stimulus parameters. (A), (B), (C) and (D) denote the correlations between the mean flight altitude, mean flight speed, and mean flight curvature of the robo-pigeon under the stimulus parameters ISI_2_+SC_3_, ISI_2_+SC_5_, ISI_3_+SC_3_, and ISI_3_+SC_5_, respectively. Abbreviations: ISI denotes the inter-stimulus interval, SC denotes the stimulus cycles, and the subscript numbers represent the duration of the inter-stimulus interval in seconds and the number of stimulus cycles, respectively. Blue ellipses indicate negative correlations, red ellipses indicate positive correlations, and the flatter the ellipse shape the stronger the correlation. H denotes mean flight altitude, V denotes mean flight speed, and C denotes mean flight curvature, where **p* < 0.05 and ***p* < 0.001.

**Fig. S2. Analysis of flight variables in eight robo-pigeons under different stimulation parameter combinations.** (A) Changes in flight altitude for eight robo-pigeons; (B) Changes in flight speed for eight robo-pigeons; (C) Changes in flight curvature for eight robo-pigeons. Abbreviations: ISI denotes the inter-stimulus interval, SC denotes the stimulus cycles, and the subscript numbers represent the duration of the inter-stimulus interval in seconds and the number of stimulus cycles, respectively. “Rp” represents robo-pigeon, and the accompanying number denotes the identifier for each individual robo-pigeon.


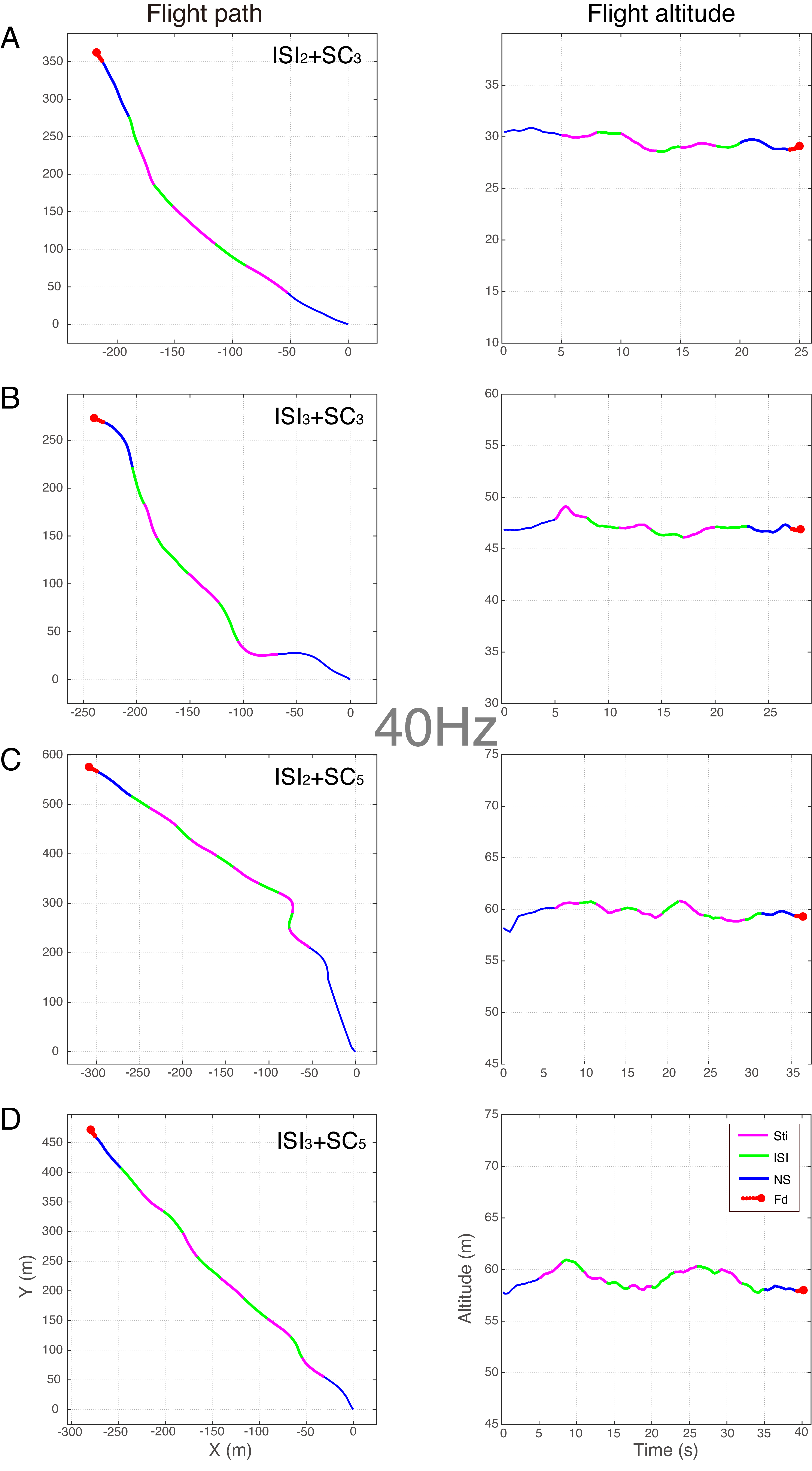
**Fig. S3.** Typical example of changes in flight trajectory (left) and flight altitude (right) of a robo-pigeon in response to 40Hz stimulation. (A), (B), (C) and (D) denote the flight trajectory and flight altitude changes of the robo-pigeon under the effect of 40 Hz stimulation at different ISIs and SCs, respectively. Abbreviations: ISI denotes the inter-stimulus interval, SC denotes the stimulus cycles, and the subscript numbers represent the duration of the inter-stimulus interval in seconds and the number of stimulus cycles, respectively. Sti, denotes stimulus segment; ISI, denotes stimulus interval; Ns, denotes non-stimulus segment; Fd, indicates flight direction.

**
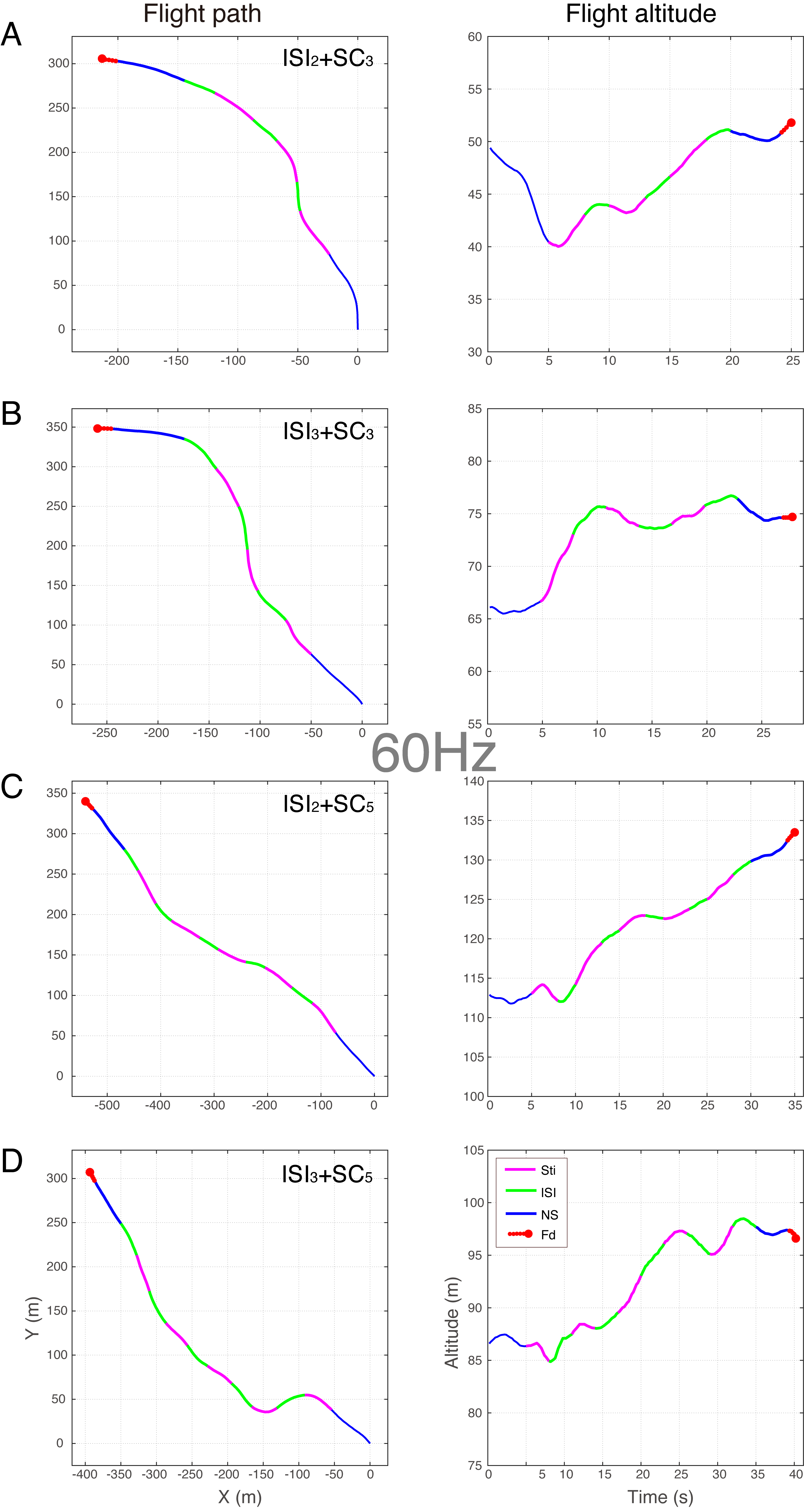
Fig. S4.** Typical example of changes in flight trajectory (left) and flight altitude (right) of a robo-pigeon in response to 60Hz stimulation. (A), (B), (C) and (D) denote the flight trajectory and flight altitude changes of the robo-pigeon under the effect of 60 Hz stimulation at different ISIs and SCs, respectively. Abbreviations: ISI denotes the inter-stimulus interval, SC denotes the stimulus cycles, and the subscript numbers represent the duration of the inter-stimulus interval in seconds and the number of stimulus cycles, respectively. Sti, denotes stimulus segment; ISI, denotes stimulus interval; Ns, denotes non-stimulus segment; Fd, indicates flight direction.


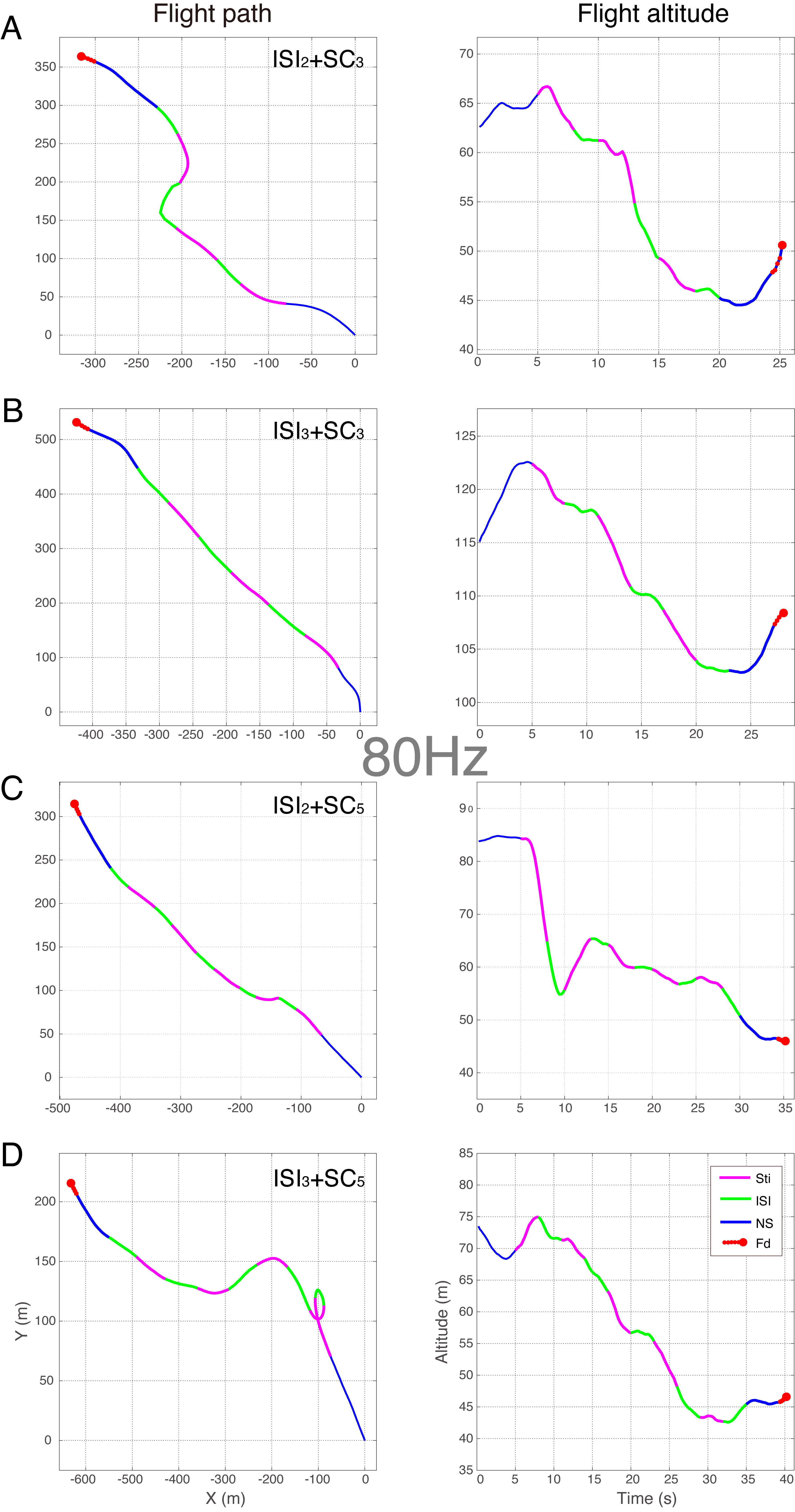
**Fig. S5.** Typical example of changes in flight trajectory (left) and flight altitude (right) of a robo-pigeon in response to 80Hz stimulation. (A), (B), (C) and (D) denote the flight trajectory and flight altitude changes of the robo-pigeon under the effect of 80 Hz stimulation at different ISIs and SCs, respectively. Abbreviations: ISI denotes the inter-stimulus interval, SC denotes the stimulus cycles, and the subscript numbers represent the duration of the inter-stimulus interval in seconds and the number of stimulus cycles, respectively. Sti, denotes stimulus segment; ISI, denotes stimulus interval; Ns, denotes non-stimulus segment; Fd, indicates flight direction.


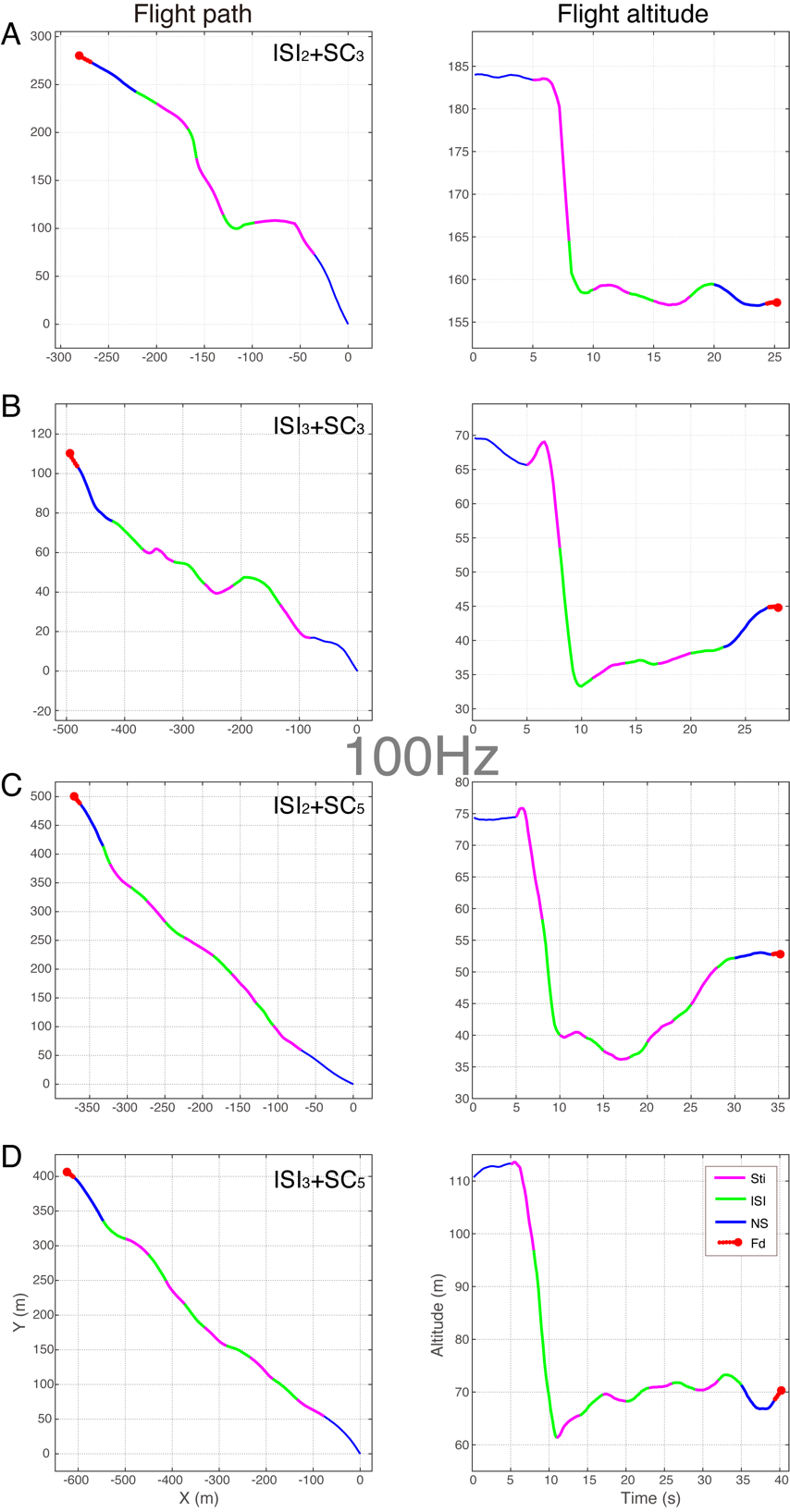
**Fig. S6.** Typical example of changes in flight trajectory (left) and flight altitude (right) of a robo-pigeon in response to 100Hz stimulation. (A), (B), (C), and (D) denote the flight trajectory and flight altitude changes of the robo-pigeon under the effect of 100 Hz stimulation at different ISIs and SCs, respectively. Abbreviations: ISI denotes the inter-stimulus interval, SC denotes the stimulus cycles, and the subscript numbers represent the duration of the inter-stimulus interval in seconds and the number of stimulus cycles, respectively. Sti, denotes stimulus segment; ISI, denotes stimulus interval; Ns, denotes non-stimulus segment; Fd, indicates flight direction.


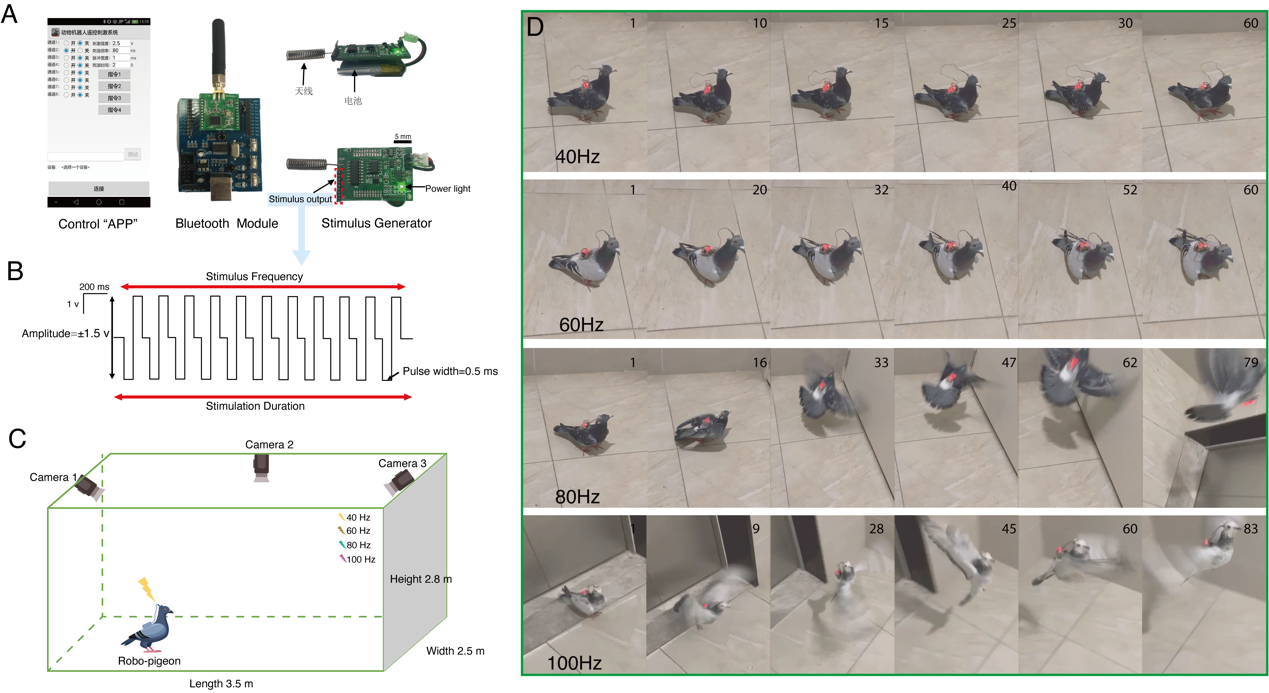
**Fig. S7. Analysis of robotic pigeons' behavioral response to loc nucleus stimulation in an indoor environment**. (A) Components of the animal robot remote stimulation system; (B) Configuration of unit stimulation pulse sequences, where red arrows indicate adjustable stimulation parameters and black arrows represent fixed parameters; (C) Schematic diagram of the indoor experimental setup used for testing the motor functions of robotic pigeons. **(D) Response characteristics of robotic pigeons' takeoff behavior under different stimulation parameters.** Note: The numbers in the figure indicate the frame number in the video recording, with the camera capturing at 30 fps.

**
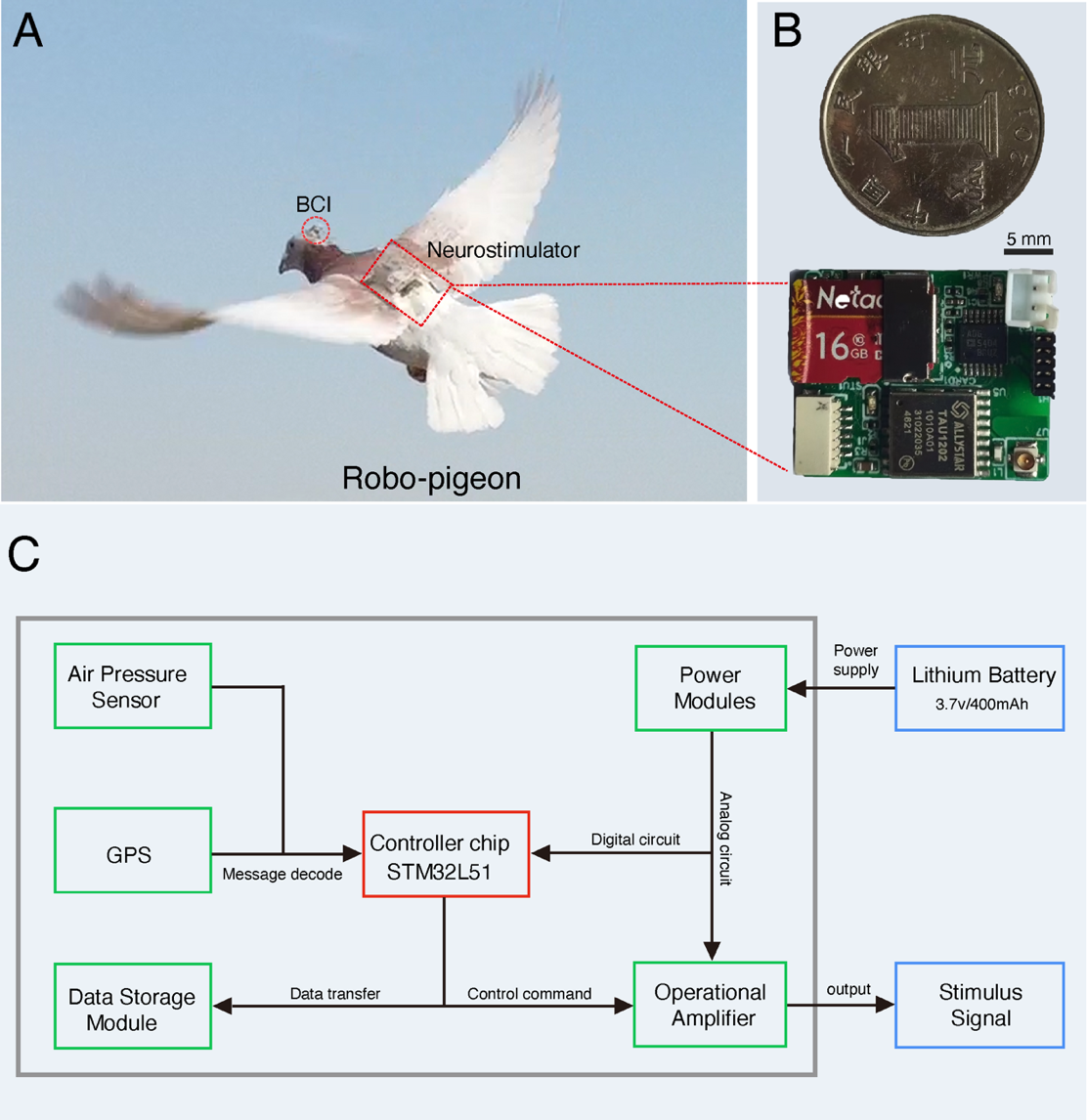
**

**Fig. S8.** The outdoor neurostimulator carried by the robo-pigeon and its system structure block diagram. (A) robo-pigeon carrying a neurostimulator on its back, (B) physical diagram of the second generation neurostimulator, (C) block diagram of the main system structure of the neurostimulator. Abbreviations: BCI, brain-computer interface.

**Table S1.** Flight eigenvalues of all pigeon robots under different stimulus parameters.

| **Variables** | **Eigenvalue** | **Pigeon_01** | **Pigeon_02** | **Pigeon_03** | **Pigeon_04** | **Pigeon_05** | **Pigeon_06** | **Pigeon_07** | **Pigeon_08** | **Mean ± SD** |
| --- | --- | --- | --- | --- | --- | --- | --- | --- | --- | --- |
| **40Hz** |  |  | | | | | | | | |
| Pre | $\bar{A}$_pre_ | 95.898±40.132 | 63.137±28.792 | 64.791±19.238 | 60.149±27.640 | 68.127±30.153 | 63.090±16.118 | 40.500±1.745 | 65.834+17.644 | 65.190±14.124 |
|  | $\bar{V}$_pre_ | 15.947±2.626 | 16.752±2.341 | 15.090±1.081 | 17.464±0.900 | 15.886±1.239 | 15.257±1.263 | 15.755±1.569 | 16.266±1.047 | 16.052±0.726 |
|  | $\bar{C}$_pre_ | 0.001±0.003 | -0.001±0.002 | 0.003±0.003 | 0.002±0.004 | 0.002±0.002 | 0.001±0.003 | 0.001±0.004 | 0.001±0.001 | 0.001±0.001 |
| ISI_2_+SC_3_ | $\bar{A}$_diff_ | 0.319±1.656 | 0.839±0.878 | 0.248±0.837 | 2.200±1.814 | 0.733±1.041 | 1.153±1.973 | 3.783±2.429 | 0.444±0.854 | 1.215±1.435 |
|  | $\bar{V}$_dur_ | 16.423±0.731 | 13.347±0.825 | 13.337±0.568 | 17.186±0.635 | 15.726±1.219 | 16.139±1.184 | 13.977±0.956 | 14.954±0.726 | 15.136±1.370 |
|  | $\bar{V}$_diff_ | -2.021±0.531 | -1.174±1.012 | -1.455±0.865 | -1.892±0.651 | -1.822±0.571 | -2.880±0.154 | -2.480±0.143 | -2.047±1.057 | -1.971±0.503 |
|  | $\bar{C}$_dur_ | 0.001±0.013 | -0.010±0.025 | 0.003±0.030 | -0.006±0.011 | -0.006±0.018 | 0.002±0.023 | 0.001±0.026 | -0.001±0.012 | -0.002±0.004 |
|  | N | 7 (0) | 7(0) | 7(0) | 7(1) | 7(0) | 7(0) | 7(0) | 7(1) | 56 (2) |
| ISI_2_+SC_5_ | $\bar{A}$_diff_ | -0.200±1.868 | 1.267±0.915 | 1.733±1.526 | -0.589±1.240 | 2.989±1.467 | -0.872±1.115 | 2.383±3.509 | 1.333±1.871 | 1.006±1.326 |
|  | $\bar{V}$_dur_ | 17.878±0.980 | 18.054±0.881 | 15.936±0.782 | 16.956±1.048 | 16.588±0.633 | 15.913±0.422 | 15.178±0.832 | 11.414±1.887 | 15.990±1.960 |
|  | $\bar{V}$_diff_ | -1.550±0.432 | -1.109±0.628 | -1.829±0.315 | -0.964±0.128 | -1.157±0.876 | -1.312±0.391 | -1.029±0.872 | -1.956±0.541 | -1.365±0.353 |
|  | $\bar{C}$_dur_ | -0.001±0.010 | -0.001±0.010 | -0.001±0.014 | -0.001±0.015 | 0.002±0.005 | 0.001±0.009 | 0.008±0.016 | 0.008±0.023 | 0.002±0.004 |
|  | N | 5 (1) | 7 (0) | 8 (0) | 7 (0) | 7 (1) | 6(0) | 7 (0) | 6 (0) | 53 (2) |
| ISI_3_+SC_3_ | $\bar{A}$_diff_ | -0.094±1.566 | 1.158±1.553 | -0.656±0.718 | 0.292±1.659 | 1.887±1.393 | 2.658±0.776 | 1.020±1.082 | 0.067±2.700 | 0.791±1.032 |
|  | $\bar{V}$_dur_ | 19.391±1.333 | 20.394±0.537 | 15.938±1.371 | 16.982±2.090 | 12.196±2.586 | 12.037±1.978 | 16.639±0.790 | 17.282±1.167 | 16.395±2.820 |
|  | $\bar{V}$_diff_ | -1.109±0.665 | -0.793±0.812 | -2.001±0.926 | -3.105±0.671 | -2.522±0.821 | -3.851±0.542 | -3.064±0.917 | -2.433±0.243 | -2.361±0.965 |
|  | $\bar{C}$_dur_ | 0.001±0.011 | -0.001±0.07 | -0.001±0.022 | -0.001±0.012 | -0.019±0.048 | -0.025±0.033 | 0.001±0.010 | 0.003±0.014 | -0.005±0.010 |
|  | N | 6 (0) | 5 (0) | 5 (0) | 5 (0) | 5 (0) | 5 (0) | 6 (0) | 8 (0) | 45 (0) |
| ISI_3_+SC_5_ | $\bar{A}$_diff_ | 1.983±1.813 | 0.858±1.450 | 0.853±1.514 | 2.567±2.217 | 1.773±1.006 | 1.233±1.205 | 0.656±1.205 | 1.367±1.252 | 1.247±0.901 |
|  | $\bar{V}$_dur_ | 10.073±0.593 | 14.647±0.962 | 14.591±1.054 | 17.764±1.683 | 16.334±0.607 | 13.357±0.452 | 15.011±1.066 | 16.766±0.951 | 14.818±2.223 |
|  | $\bar{V}$_diff_ | -1.967±0.234 | -1.517±0.672 | -3.982±1.382 | -3.561±1.652 | -0.407±0.961 | -2.163±0.132 | -1.605±0.776 | -1.912±1.002 | -2.139±1.071 |
|  | $\bar{C}$_dur_ | 0.001±0.022 | 0.002±0.013 | -0.012±0.019 | -0.001±0.017 | -0.001±0.011 | -0.006±0.017 | -0.006±0.024 | 0.007±0.019 | -0.002±0.005 |
|  | N | 5 (0) | 5 (0) | 6 (0) | 6 (1) | 7 (0) | 7 (0) | 6 (0) | 5 (0) | 47 (1) |
| Post | $\bar{A}$_post_ | 94.356±39.781 | 62.604±27.947 | 64.728±19.303 | 61.367±25.421 | 70.253±30.756 | 63.717±17.153 | 40.282±8.841 | 66.776±17.783 | 65.510±13.794 |
|  | $\bar{V}$_post_ | 16.312±3.382 | 17.084±2.970 | 14.885±1.181 | 17.45±0.577 | 16.083±0.846 | 15.076±1.603 | 16.014±0.869 | 14.653±3.544 | 15.945±0.953 |
|  | $\bar{C}$_post_ | 0.003±0.004 | -0.004±0.004 | 0.003±0.001 | 0.001±0.003 | 0.001±0.001 | 0.005±0.004 | -0.001±0.003 | 0.006±0.017 | 0.002±.003 |
| **60Hz** |  |  |  |  |  |  |  |  |  |  |
| Pre | $\bar{A}$_pre_ | 107.301±20.581 | 68.836±23.240 | 62.813±24.587 | 76.161±24.147 | 41.499±18.044 | 60.385±13.178 | 55.536±8.715 | 43.513±13.980 | 64.505±19.546 |
|  | $\bar{V}$_pre_ | 18.430±3.111 | 16.762±2.360 | 15.349±0.907 | 18.924±3.458 | 20.622±6.706 | 18.042±2.367 | 17.983±1.244 | 16.083±1.095 | 17.774±1.571 |
|  | $\bar{C}$_pre_ | 0.004±0.001 | 0.015±0.022 | 0.005±0.004 | 0.002±0.003 | -0.004±0.008 | -0.017±0.016 | -0.002±0.002 | -0.004±0.005 | -0.001±0.009 |
| ISI_2_+SC_3_ | $\bar{A}$_diff_ | 9.921±2.918 | 10.950±3.347 | 10.227±5.367 | 9.170±2.930 | 10.270±3.306 | 11.441±3.235 | 12.364±3.922 | 11.914±3.403 | 10.782±1.010 |
|  | $\bar{V}$_dur_ | 15.495±0.624 | 16.136±1.064 | 12.875±1.152 | 23.072±1.152 | 15.242±1.051 | 15.783±1.892 | 17.263±1.158 | 17.150±0.971 | 16.627±1.178 |
|  | $\bar{V}$_diff_ | -3.372±0.916 | -4.839±1.068 | -3.425±2.004 | -2.201±1.083 | -4.813±2.413 | -3.527±1.921 | -3.326±1.723 | -4.794±0.752 | -3.832±0.872 |
|  | $\bar{C}$_dur_ | -0.002±0.1022 | 0.002±0.012 | 0.0258±0.757 | -0.001±0.006 | -0.012±0.018 | 0.017±0.337 | -0.001±0.006 | 0.002±0.011 | 0.004±0.011 |
|  | N | 13 (11) | 10 (8) | 11 (10) | 10 (10) | 10 (9) | 12 (9) | 13 (11) | 9 (7) | 88 (75) |
| ISI_2_+SC_5_ | $\bar{A}$_diff_ | 13.333±3.775 | 10.275±3.226 | 13.700±4.806 | 14.889±4.189 | 8.342±3.806 | 10.953±3.855 | 15.500±4.608 | 11.928±2.065 | 12.365±2.282 |
|  | $\bar{V}$_dur_ | 15.418±1.469 | 15.532±1.016 | 14.707±1.244 | 16.261±1.903 | 15.078±0.863 | 12.920±1.729 | 14.186±0.981 | 13.401±1.317 | 14.688±1.055 |
|  | $\bar{V}$_diff_ | -6.257±2.523 | -3.754±1.993 | -5.877±3.003 | -5.252±2.334 | -2.374±0.873 | -6.112±2.651 | -4.236±1.965 | -5.295±0.173 | -4.896±1.259 |
|  | $\bar{C}$_dur_ | -0.01±0.011 | -0.002±0.014 | 0.003±0.019 | -0.001±0.014 | -0.004±0.015 | -0.006±0.018 | -0.005±0.017 | 0.002±0.010 | -0.002±0.003 |
|  | N | 5 (5) | 6 (5) | 7 (6) | 8 (7) | 10 (8) | 7 (6) | 7 (7) | 9 (8) | 59 (52) |
| ISI_3_+SC_3_ | $\bar{A}$_diff_ | 10.931±2.717 | 9.041±2.605 | 10.937±2.660 | 11.062±3.444 | 8.606±2.068 | 10.560±2.911 | 12.342±3.633 | 12.487±2.535 | 10.745±1.288 |
|  | $\bar{V}$_dur_ | 16.279±1.573 | 16.714±1.800 | 12.744±1.899 | 16.164±1.398 | 15.865±1.829 | 13.159±0.659 | 16.717±1.208 | 14.036±2.460 | 15.210±1.528 |
|  | $\bar{V}$_diff_ | -4.537± | -6.920± | -3.579± | -6.674± | -8.413± | -3.783± | -8.358± | -9.062± | -6.416±2.049 |
|  | $\bar{C}$_dur_ | -0.002±0.010 | 0.001±0.008 | -0.012±0.043 | -0.001±0.012 | -0.002±0.014 | -0.013±0.030 | 0.001±0.007 | 0.028±0.055 | 0.001±0.012 |
|  | N | 15 (13) | 10 (9) | 10 (9) | 10 (7) | 8 (7) | 15 (13) | 10 (8) | 7 (7) | 85 (73) |
| ISI_3_+SC_5_ | $\bar{A}$_diff_ | 18.600±2.527 | 13.847±4.702 | 14.533±3.423 | 12.658±3.538 | 15.325±4.532 | 12.522±5.124 | 17.308±3.562 | 15.789±3.695 | 15.073±2.005 |
|  | $\bar{V}$_dur_ | 18.425±2.095 | 15.918±1.025 | 15.876±1.231 | 16.076±1.186 | 14.263±1.043 | 16.987±1.232 | 18.880±1.084 | 16.921±1.073 | 16.771±1.358 |
|  | $\bar{V}$_diff_ | -7.348±2.112 | -6.053±0.928 | -4.710±0.916 | -7.436±2.867 | -6.808±1.549 | -6.191±2.933 | -6.011±1.544 | -9.433±1.192 | -6.749±1.301 |
|  | $\bar{C}$_dur_ | 0.006±0.016 | 0.001±0.069 | 0.001±0.024 | -0.004±0.018 | -0.004±0.090 | -0.002±0.012 | 0.012±0.169 | 0.003±0.031 | 0.002±0.005 |
|  | N | 8 (6) | 8 (6) | 7 (6) | 5 (5) | 5 (5) | 9 (8) | 6 (6) | 7 (7) | 55 (49) |
| Post | $\bar{A}$_post_ | 119.229±22.437 | 75.929±23.960 | 72.261±26.556 | 85.192±27.605 | 51.672±17.221 | 69.994±13.942 | 64.773±11.406 | 51.812±13.728 | 73.858±13.585 |
|  | $\bar{V}$_post_ | 16.976±0.821 | 15.978±2.357 | 19.236±6.310 | 17.254±3.106 | 15.988±0.693 | 16.974±1.232 | 17.271±0.776 | 15.014±3.237 | 16.836±1.169 |
|  | $\bar{C}$_post_ | 0.001±0.002 | 0.001±0.003 | 0.002±0.004 | 0.001±0.001 | 0.002±0.003 | -0.003±0.001 | 0.001±0.003 | 0.004±0.011 | 0.001±0.002 |
| **80Hz** |  |  |  |  |  |  |  |  |  |  |
| Pre | $\bar{A}$_pre_ | 118.512±7.452 | 70.906±5.798 | 54.164±11.193 | 79.752±11.576 | 63.500±9.677 | 67.746±21.084 | 64.419±1.669 | 58.424±4.803 | 74.143±18.229 |
|  | $\bar{V}$_pre_ | 17.263±0.798 | 17.208±0.481 | 18.028±1.161 | 18.318±2.076 | 17.290±3.103 | 20.755±6.117 | 17.311±1.736 | 17.667±2.671 | 17.980±1.115 |
|  | $\bar{C}$_pre_ | 0.004±0.006 | 0.001±0.005 | -0.003±0.004 | 0.004±0.005 | -0.005±0.005 | -0.008±0.001 | 0.001±0.001 | 0.004±0.002 | -0.001±0.004 |
| ISI_2_+SC_3_ | $\bar{A}$_diff_ | -10.750±3.665 | -17.346±5.166 | -10.633±3.342 | -13.114±4.705 | -11.139±2.614 | -10.792±3.880 | -21.543±7.229 | -12.217±3.754 | -13.442±3.707 |
|  | $\bar{V}$_dur_ | 17.310±1.833 | 16.762±1.693 | 19.307±4.785 | 21.996±1.195 | 18.628±0.370 | 16.164±1.518 | 18.824±1.194 | 19.135±4.206 | 18.516±1.706 |
|  | $\bar{V}$_diff_ | 7.575±2.722 | 3.500±0.721 | 7.132±2.241 | 5.446±1.962 | 1.560±0.275 | 3.093±0.986 | 3.857±1.151 | 7.565±2.553 | 4.966±2.150 |
|  | $\bar{C}$_dur_ | 0.007±0.020 | -0.023±0.037 | -0.003±0.015 | -0.007±0.017 | -0.004±0.010 | -0.004±0.018 | -0.010±0.054 | 0.002±0.045 | -0.005±0.008 |
|  | N | 13 (10) | 15 (13) | 15 (14) | 20 (17) | 12 (11) | 7 (7) | 12 (11) | 15 (14) | 109 (97) |
| ISI_2_+SC_5_ | $\bar{A}$_diff_ | -20.725±5.558 | -14.410±7.705 | -17.450±6.736 | -19.900±5.706 | -16.039±4.149 | -15.707±5.412 | -16.446±5.410 | -12.012±3.063 | -16.586±2.636 |
|  | $\bar{V}$_dur_ | 18.918±1.738 | 17.058±1.579 | 18.323±1.872 | 17.0687±1.734 | 15.325±2.027 | 17.138±2.149 | 15.308±2.761 | 14.540±2.261 | 16.710±1.436 |
|  | $\bar{V}$_diff_ | 6.090± | 6.992± | 5.907± | 6.829± | 5.916± | 6.518± | 7.762± | 4.241± | 6.282±0.970 |
|  | $\bar{C}$_dur_ | 0.003±0.007 | 0.001±0.025 | -0.001±0.056 | -0.001±0.013 | -0.002±0.010 | -0.0034±0.008 | -0.008±0.020 | 0.003±0.014 | -0.001±0.003 |
|  | N | 8 (8) | 8 (7) | 8 (8) | 8 (7) | 8 (6) | 10 (9) | 15 (13) | 12 (11) | 77 (69) |
| ISI_3_+SC_3_ | $\bar{A}$_diff_ | -12.830±4.672 | -11.279±4.170 | -14.200±4.203 | -12.276±3.631 | -12.100±3.607 | -11.717±3.678 | -19.448±5.782 | -12.029±6.500 | -13.235±2.487 |
|  | $\bar{V}$_dur_ | 18.567±1.551 | 18.550±0.702 | 16.892±4.502 | 17.474±2.755 | 17.826±2.346 | 14.759±2.485 | 16.439±2.953 | 15.719±3.386 | 17.028±1.262 |
|  | $\bar{V}$_diff_ | 3.231±1.243 | 1.815±0.976 | 6.561±2.881 | 5.891±2.615 | 7.271±2.995 | 11.923±3.471 | 6.154±1.876 | 10.608±2.965 | 6.682±3.157 |
|  | $\bar{C}$_dur_ | 0.004±0.012 | 0.001±0.009 | -0.004±0.041 | 0.001±0.012 | -0.003±0.009 | -0.007±0.020 | -0.006±0.006 | 0.020±0.045 | 0.001±0.008 |
|  | N | 12 (11) | 12 (11) | 10 (8) | 11 (11) | 15 (13) | 10 (10) | 10 (9) | 16 (16) | 96 (89) |
| ISI_3_+SC_5_ | $\bar{A}$_diff_ | -22.100±4.608 | -20.646±6.172 | -18.719±6.282 | -18.676±4.461 | -17.706±4.696 | -19.742±3.736 | -20.118±5.082 | -17.144±3.922 | -19.356±1.517 |
|  | $\bar{V}$_dur_ | 16.378±5.519 | 17.618±1.417 | 17.269±1.683 | 14.057±2.083 | 12.699±0.826 | 13.988±1.341 | 15.138±1.892 | 13.315±2.161 | 15.058±1.730 |
|  | $\bar{V}$_diff_ | 10.589±4.489 | 5.196±2.182 | 3.953±0.915 | 4.313±1.790 | 2.416±1.171 | 6.469±2.749 | 10.283±3.741 | 10.640±3.378 | 6.732±3.110 |
|  | $\bar{C}$_dur_ | 0.001±0.018 | -0.001±0.016 | 0.002±0.039 | 0.003±0.011 | -0.005±0.011 | -0.013±0.021 | -0.005±0.043 | 0.002±0.019 | -0.002±0.005 |
|  | N | 10 (8) | 8 (8) | 10 (9) | 8 (7) | 12 (12) | 9 (8) | 15 (13) | 8 (7) | 80 (72) |
| Post | $\bar{A}$_post_ | 101.861±12.017 | 54.180±7.169 | 39.196±9.421 | 63.677±13.561 | 49.419±8.763 | 54.276±19.537 | 45.128±16.483 | 45.753±5.128 | 56.686±18.417 |
|  | $\bar{V}$_post_ | 18.540±1.767 | 16.833±0.719 | 15.230±2.683 | 18.336±1.607 | 16.857±1.407 | 19.360±3.370 | 18.085±3.145 | 16.295±1.505 | 17.442±1.276 |
|  | $\bar{C}$_post_ | 0.001±0.003 | -0.002±0.002 | 0.001±0.002 | -0.001±0.001 | -0.001±0.002 | -0.003±0.003 | -0.005±0.009 | 0.001±0.003 | -0.001±0.002 |
| **100Hz** |  |  |  |  |  |  |  |  |  |  |
| Pre | $\bar{A}$_pre_ | 95.505±21.239 | 78.010±31.214 | 99.249±10.321 | 67.064±14.930 | 59.801±20.521 | NA | 73.298±17.081 | 65.768±12.530 | 76.956±14.004 |
|  | $\bar{V}$_pre_ | 17.847±1.742 | 19.887±1.172 | 17.97±1.502 | 20.143±3.672 | 17.109±1.273 | NA | 17.640±1.115 | 17.246±1.147 | 18.264±1.146 |
|  | $\bar{C}$_pre_ | -0.001±0.003 | 0.001±0.003 | 0.004±0.002 | 0.002±0.002 | -0.002±0.003 | NA | 0.001±0.001 | -0.001±0.003 | 0.001±0.002 |
| ISI_2_+SC_3_ | $\bar{A}$_diff_ | -17.111±8.969 | -23.767±16.030 | NA | -20.217±8.719 | -24.767±7.726 | NA | -17.067±10.667 | -21.167±11.201 | -20.682±2.957 |
|  | $\bar{V}$_dur_ | 14.953±3902 | 17.787±3.079 | NA | 24.195±3.108 | 16.611±2.646 | NA | 16.581±3.026 | 19.086±4.327 | 18.202±2.960 |
|  | $\bar{V}$_diff_ | 11.525±4.713 | 4.595±1.124 | NA | 4.736±0.768 | 12.954±4.736 | NA | 7.867±1.096 | 11.468±3.063 | 8.857±3.337 |
|  | $\bar{C}$_dur_ | 0.003±0.040 | 0.004±0.045 | NA | -0.029±0.085 | -0.005±0.045 | NA | 0.015±0.063 | 0.001±0.030 | -0.002±0.014 |
|  | N | 5 (5) | 5 (5) | NA | 6 (6) | 7 (7) | NA | 6 (6) | 5 (5) | 34 (34) |
| ISI_2_+SC_5_ | $\bar{A}$_diff_ | -23.900±9.731 | -45.444±9.046 | NA | -22.700±4.039 | -22.378±6.825 | NA | -28.775±7.127 | -34.156±7.977 | -29.559±8.213 |
|  | $\bar{V}$_dur_ | 16.699±2.397 | 16.833±2.681 | NA | 15.974±1.946 | 16.201±1.864 | NA | 15.956±3.056 | 10.868±4.827 | 15.422±2.064 |
|  | $\bar{V}$_diff_ | 11.178±3.152 | 13.979±3.866 | NA | 9.146±2.711 | 8.011±0.052 | NA | 10.177±3.338 | 14.154±3.364 | 10.146±0.818 |
|  | $\bar{C}$_dur_ | -0.001±0.026 | 0.001±0.020 | NA | -0.002±0.018 | -0.002±0.021 | NA | -0.001±0.021 | -0.0123±0.047 | -0.003±0.004 |
|  | N | 7 (7) | 5 (5) | NA | 5 (5) | 6 (6) | NA | 7 (7) | 8 (8) | 38 (38) |
| ISI_3_+SC_3_ | $\bar{A}$_diff_ | -39.483±16.894 | -26.7667±13.866 | -26.456±14.424 | -32.133±13.003 | -27.533±6.337 | NA | -28.800±7.573 | -28.233±10.756 | -29.915±4.278 |
|  | $\bar{V}$_dur_ | 19.154±3.629 | 21.585±2.761 | 20.430±3.168 | 16.499±4.526 | 14.905±4.811 | NA | 14.000±4.073 | 19.488±2.004 | 18.009±2.676 |
|  | $\bar{V}$_diff_ | 8.703±1.697 | 6.018±2.926 | 10.405±3.281 | 10.664±3.705 | 11.309±4.037 | NA | 9.978±1.077 | 14.154±4.239 | 10.176±2.296 |
|  | $\bar{C}$_dur_ | 0.002±0.025 | 0.001±0.016 | -0.003±0.026 | 0.005±0.050 | -0.00±0.072 | NA | 0.001±0.056 | -0.001±0.012 | -0.0001±0.004 |
|  | N | 7 (7) | 7 (7) | 6 (6) | 8 (8) | 5 (5) | NA | 5 (5) | 5 (5) | 43 (43) |
| ISI_3_+SC_5_ | $\bar{A}$_diff_ | -37.850±11.984 | -38.667±8.150 | NA | -37.433±8.704 | -44.633±1.963 | NA | -30.450±7.152 | -30.458±7.376 | -36.582±4.943 |
|  | $\bar{V}$_dur_ | 18.552±1.909 | 18.506±2.739 | NA | 16.530±4.477 | 14.413±1.126 | NA | 16.992±1.912 | 16.770±2.628 | 16.961±1.393 |
|  | $\bar{V}$_diff_ | 10.274±3.283 | 9.746±2.966 | NA | 15.031±5.816 | 7.593±1.056 | NA | 9.287±2.906 | 10.725±4.617 | 10.443±2.277 |
|  | $\bar{C}$_dur_ | 0.001±0.013 | 0.001±0.021 | NA | 0.004±0.028 | 0.001±0.016 | NA | 0.001±0.014 | 0.001±0.009 | 0.001±0.001 |
|  | N | 5 (5) | 5 (5) | NA | 6 (6) | 7 (7) | NA | 5 (5) | 7 (7) | 35 (35) |
| Post | $\bar{A}$_post_ | 65.376±19.876 | 45.261±26.761 | 60.832±21.437 | 44.474±22.431 | 47.347±29.730 | NA | 52.673±19.874 | 41.990±10.904 | 51.136±8.247 |
|  | $\bar{V}$_post_ | 15.449±2.637 | 17.479±1.523 | 19.116±1.385 | 17.838±3.736 | 14.528±0.954 | NA | 16.300±1.879 | 15.925±4.494 | 16.662±1.453 |
|  | $\bar{C}$_post_ | 0.007±0.015 | 0.004±0.012 | 0.001±0.011 | 0.001±0.005 | 0.001±0.003 | NA | 0.003±0.004 | 0.001±0.005 | 0.003±0.002 |

**Note:** ISI_2_+SC_3_ denotes the inter-stimulation interval of 2 s and 3 stimulation cycles; ISI_2_+SC_5_ denotes the inter-stimulation interval of 2 s and 5 stimulation cycles; ISI_3_+SC_3_ denotes the inter-stimulation interval of 3 s and 3 stimulation cycles; ISI_3_+SC_5_ denotes the inter-stimulation interval of 3 s and 5 stimulation cycles. t_pre_ denotes the first 5 s of the stimulus segment; t_post_ denotes the post-stimulus 5 s of the stimulus segment; $\bar{V}$_pre_ denotes the mean flight speed during the t_pre_, $\bar{C}$_pre_ denotes the mean flight curvature during the t_pre_, $\bar{A}$_pre_ denotes the mean flight altitude during the t_pre_; $\bar{V}$_dur_ denotes mean flight speed during the stimulus, $\bar{C}$_dur_ denotes mean flight curvature during the stimulus, $\bar{V}$_diff_ denotes the maximum difference in the change of its flight speed during the stimulus; $\bar{A}$_diff_ denotes the difference in flight altitude before and after the stimulus; $\bar{V}$_post_ denotes mean flight speed during the t_post_, $\bar{C}$_post_ denotes the mean flight curvature during t_post_ time, and $\bar{V}$_post_ denotes the mean flight altitude during t_post_.

**Table S2.** Results of AVOVA analysis of individual flight variables in pigeon robots under different stimulus parameters.

| Factor | Ascending | | | | |  | Descending | | | | | | | |
| --- | --- | --- | --- | --- | --- | --- | --- | --- | --- | --- | --- | --- | --- | --- |
|  | *F* | *ε* | *p* | Partial *η^2^* | LSD |  | *F* | *ε* | *p* | Partial *η^2^* | | | LSD | |
| $\bar{\boldsymbol{A}}$**_diff_** |  |  |  |  |  |  |  |  |  |  | | |  | |
| SF _(1 14)_ | 801.892 | 1.000 | 0.000^**^ | 0.983 | 60 Hz > 40 Hz |  | 73.789 | 1.000 | 0.000^**^ | 0.881 | | | 100 Hz > 80 Hz | |
| ISI _(1 14)_ | 2.468 | NA | 0.139 | 0.150 | NA |  | 12.073 | NA | 0.006^**^ | 0.547 | | | ISI_3_ > ISI_2_ | |
| SC _(1 14)_ | 31.030 | 1.000 | 0.000^**^ | 0.689 | SC_5_>SC_3_ |  | 30.929 | 1.000 | 0.000^**^ | 0.756 | | | SC_5_>SC_3_ | |
| SF * ISI _(1 14)_ | 3.533 | NA | 0.081 | 0.202 | NA |  | 5.805 | NA | 0.037^*^ | 0.367 | | | See Table S3 | |
| ISI * SC _(1 14)_ | 6.387 | NA | 0.024^*^ | 0.313 | See Table 2 |  | 0.063 | NA | 0.807 | 0.006 | | | NA | |
| SF * SC _(1 14)_ | 11.461 | 1.000 | 0.004^**^ | 0.450 |  |  | 1.159 | 1.000 | 0.307 | 0.104 | | | NA | |
| SF * ISI * SC _(1 14)_ | 2.614 | NA | 0.128 | 0.157 | NA |  | 1.146 | NA | 0.310 | 0.103 | | | NA | |
| $\bar{\boldsymbol{V}}$**_diff_** |  |  |  |  |  |  |  |  |  |  | | |  | |
| SF _(1 14)_ | 92.906 | 1.000 | 0.000^**^ | 0.869 | 60 Hz > 40 Hz |  | 35.671 | 1.000 | 0.000^**^ | 0.781 | | | 100 Hz > 80 Hz | |
| ISI _(1 14)_ | 20.740 | NA | 0.000^**^ | 0.579 | ISI_3_ > ISI_2_ |  | 0.809 | NA | 0.390 | 0.075 | | | NA | |
| SC _(1 14)_ | 0.224 | 1.000 | 0.643 | 0.016 | NA |  | 1.068 | 1.000 | 0.326 | 0.097 | | | NA | |
| ISI * SC _(1 14)_ | 0.083 | NA | 0.778 | 0.006 | NA |  | 0.032 | NA | 0.861 | 0.003 | | | NA | |
| SF * ISI _(1 14)_ | 5.037 | NA | 0.041^*^ | 0.265 | See Table S3 |  | 0.000 | 1.000 | 0.991 | 0.000 | | | NA | |
| SF * SC _(1 14)_ | 4.826 | 1.000 | 0.045^*^ | 0.256 |  |  | 0.138 | 1.000 | | | 0.718 | 0.014 | | NA |
| SF * ISI * SC _(1 14)_ | 1.213 | NA | 0.289 | 0.080 | NA |  | 0.039 | NA | | | 0.848 | 0.004 | | NA |
| $\bar{\boldsymbol{C}}$**_dur_** |  |  |  |  |  |  |  |  | | |  |  | |  |
| SF _(1 14)_ | 1.207 | 1.000 | 0.290 | 0.079 | NA |  | 0.945 | 1.000 | | | 0.354 | 0.086 | | NA |
| ISI _(1 14)_ | 0.499 | NA | 0.492 | 0.034 | NA |  | 0.388 | NA | | | 0.547 | 0.037 | | NA |
| SC _(1 14)_ | 0.847 | 1.000 | 0.373 | 0.057 | NA |  | 1.115 | 1.000 | | | 0.316 | 0.100 | | NA |
| SF * ISI _(1 14)_ | 5.072 | NA | 0.541 | 0.266 | NA |  | 0.085 | 1.000 | | | 0.777 | 0.008 | | NA |
| ISI * SC _(1 14)_ | 0.392 | NA | 0.542 | 0.027 | NA |  | 1.064 | NA | | | 0.327 | 0.096 | | NA |
| SF * SC _(1 14)_ | 0.003 | 1.000 | 0.955 | 0.000 | NA |  | 0.093 | 1.000 | | | 0.767 | 0.009 | | NA |
| SF * ISI * SC _(1 14)_ | 0.672 | NA | 0.426 | 0.046 | NA |  | 0.835 | NA | | | 0.328 | 0.077 | | NA |

Note: ">" indicates that the value of the variable in the corresponding condition on its left side is greater than on the right side. The symbol "*" indicates an interaction between the factors of the factor. Degrees of freedom are shown after each factor. Abbreviations: SF indicates stimulus frequency, ISI indicates inter-stimulus interval, SC indicates stimulus cycles, and the subscript numbers represent the duration of the inter-stimulus interval in seconds and the number of stimulus cycles, respectively. N/A is not applicable. **P* < 0.05, ***P* < 0.001.

**Table S3.** Results of simple-simple effects analysis between factors in different flight variables.

| **Flight variables** | **Factor** | ***F*** | ***p*** | **Partial *η^2^*** | **LSD** |
| --- | --- | --- | --- | --- | --- |
| $\bar{\boldsymbol{A}}$**_diff_** | **Ascending** |  |  |  |  |
|  | ISI * SC _(1, 14)_ \| (ISI_2_ * SC) | 4.630 | 0.049^*^ | 0.249 | SC_5_ >SC_3_ |
|  | ISI * SC _(1, 14)_ \| (ISI_3_ * SC) | 32.787 | 0.000^**^ | 0.701 | SC_5_ >SC_3_ |
|  | SC * ISI _(1, 14)_ \| (SC_3_ * ISI) | 0.120 | 0.734 | 0.009 | NA |
|  | SC * ISI _(1, 14)_ \| (SC_5_ * ISI) | 5.756 | 0.031^*^ | 0.291 | ISI_3_ > ISI_2_ |
|  | SF * SC _(1, 14)_ \| (40 Hz * SC) | 0.957 | 0.344 | 0.064 | NA |
|  | SF * SC _(1, 14)_ \| (60 Hz * SC) | 23.632 | 0.000^**^ | 0.628 | SC_5_ >SC_3_ |
|  | SC * SF _(1, 14)_ \| (SC_3_ * SF) | 516.564 | 0.000^**^ | 0.974 | 60 Hz > 40 Hz |
|  | SC * SF _(1, 14)_ \| (SC_5_* SF) | 357.661 | 0.000^**^ | 0.962 | 60 Hz > 40 Hz |
|  | **Descending** |  |  |  |  |
|  | SF * ISI _(1, 14)_ \| (80 Hz * ISI) | 0.501 | 0.495 | 0.048 | NA |
|  | SF * ISI _(1, 14)_ \| (100 Hz * ISI) | 10.302 | 0.009^**^ | 0.507 | ISI_3_ > ISI_2_ |
|  | ISI* SF _(1, 14)_ \| (ISI_2_ * SF) | 19.101 | 0.001^**^ | 0.656 | 100 Hz > 80 Hz |
|  | ISI * SF _(1, 14)_ \| (ISI_3_ * SF) | 60.4492 | 0.000^**^ | 0.858 | 100 Hz > 80 Hz |
| $\bar{\boldsymbol{V}}$**_diff_** | **Ascending** |  |  |  |  |
|  | SF * ISI _(1, 14)_ \| (40 Hz * ISI) | 3.563 | 0.080 | 0.203 | NA |
|  | SF * ISI _(1, 14)_ \| (60 Hz * ISI) | 13.676 | 0.002^**^ | 0.494 | ISI_3_ > ISI_2_ |
|  | ISI* SF _(1, 14)_ \| (ISI_2_ * SF) | 27.339 | 0.000^**^ | 0.661 | 60 Hz > 40 Hz |
|  | ISI * SF _(1, 14)_ \| (ISI_3_ * SF) | 70.604 | 0.000^**^ | 0.835 | 60 Hz > 40 Hz |
|  | SF * SC _(1, 14)_ \| (40 Hz * SC) | 2.137 | 0.166 | 0.132 | NA |
|  | SF * SC _(1, 14)_ \| (60 Hz * SC) | 2.135 | 0.166 | 0.132 | NA |
|  | SC * SF _(1, 14)_ \| (SC_3_ * SF) | 39.275 | 0.000^**^ | 0.737 | SC_5_ >SC_3_ |
|  | SC * SF _(1, 14)_ \| (SC_5_* SF) | 96.649 | 0.000^**^ | 0.873 | SC_5_ >SC_3_ |

Note: ">" indicates that the value of the variable in the corresponding condition on its left side is greater than on the right side. The symbol "*" indicates an interaction between the factors of the factor. Degrees of freedom are shown after each factor. Abbreviations: SF indicates stimulus frequency, ISI indicates inter-stimulus interval, SC indicates stimulus cycles, and the subscript numbers represent the duration of the inter-stimulus interval in seconds and the number of stimulus cycles, respectively. N/A is not applicable. **P* < 0.05, ***P* < 0.001.

**Table S4.** Behavioral response results of pigeon LoC nuclei in response to different electrical stimulation parameters (N=15).

| Stimulation parameters | | |  | | Behavioral response | | |
| --- | --- | --- | --- | --- | --- | --- | --- |
| SV, PD | SF | SD |  | Non-responsive | | Ready take-off | Take-off |
| ±1.5 V,  0.5 ms | 40 Hz | 1 s |  | **+** | |  |  |
|  |  | 2 s |  | **+** | |  |  |
|  |  | 3 s |  | **+** | |  |  |
|  | 60 Hz | 1 s |  |  | | **+** |  |
|  |  | 2 s |  |  | | **++** |  |
|  |  | 3 s |  |  | | **+++** |  |
|  | 80 Hz | 1 s |  |  | |  | **-** |
|  |  | 2 s |  |  | |  | **+** |
|  |  | 3 s |  |  | |  | **++** |
|  | 100 Hz | 1 s |  |  | |  | **+++** |
|  |  | 2 s |  |  | |  | **+++** |
|  |  | 3 s |  |  | |  | **+++** |

**Note:** "Ready take-off" means that the pigeons showed slightly spread wings and crouched down after stimulation; " Take-off" means that the pigeons spread their wings and flew normally after stimulation. The symbols "-" and "+" indicate the intensity of the behavioral response. In the behavioral state of takeoff, "-" indicates that the pigeon's takeoff height is less than 1 m after being stimulated, and "+" indicates that the pigeon's takeoff height is more than 1 m after being stimulated, and "+" indicates that the more violent the takeoff response. The more "+", the more intense the take-off response.
