# Supplementary figures and images for: Dynamically Controlled Flight Altitudes in Robo-Pigeons via Locus Coeruleus Neurostimulation
Source: Research (Wash D C). 2025 Mar 5;8:0632. doi: 10.34133/research.0632 (PMC11880575; doi:10.34133/research.0632)

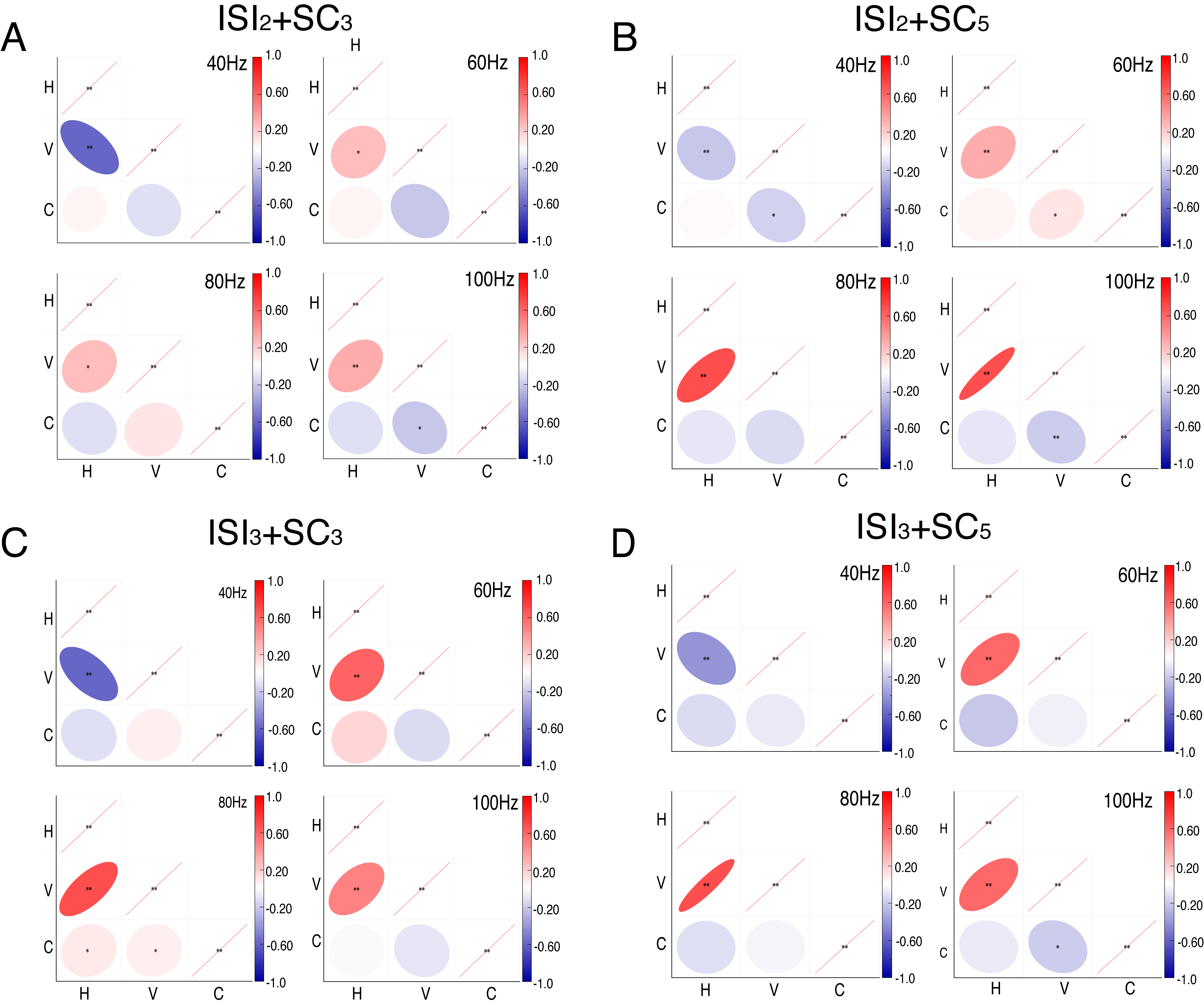

Supplement: Supplementary 1 — Figs. S1 to S8 Tables S1 to S4 [file research.0632.f1.zip › Fig. S1.tif]

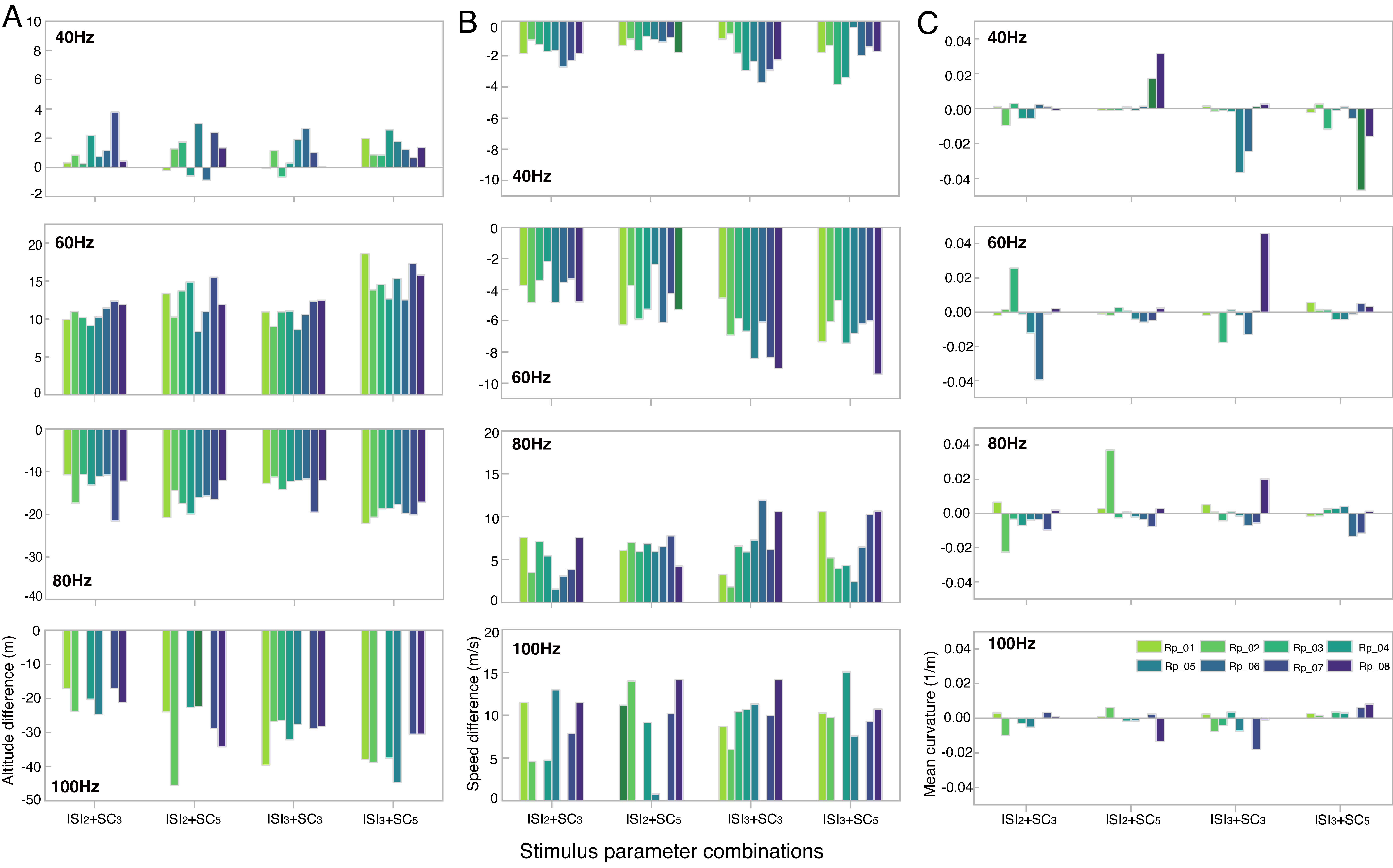

Supplement: Supplementary 1 — Figs. S1 to S8 Tables S1 to S4 [file research.0632.f1.zip › Fig. S2.tif]

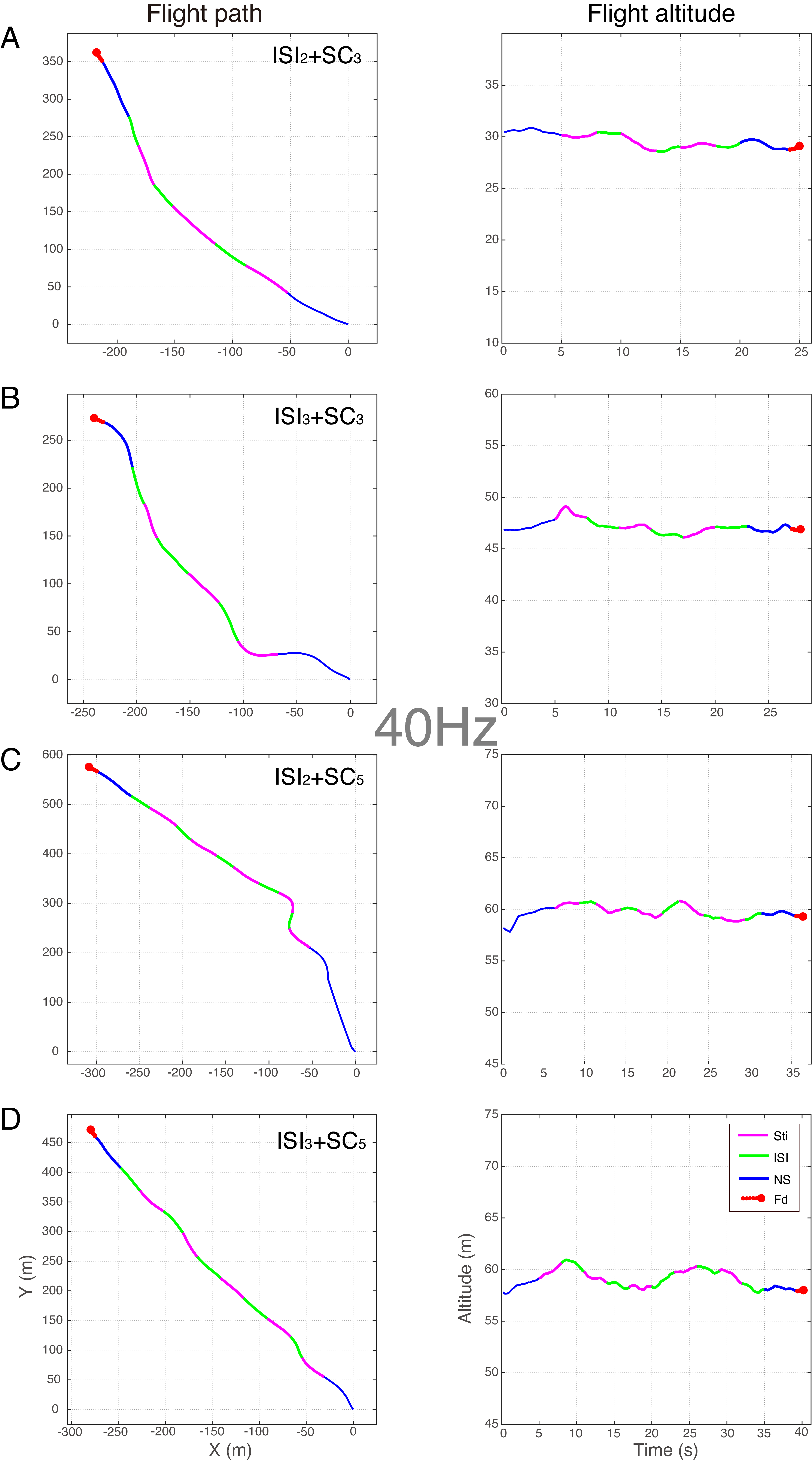

Supplement: Supplementary 1 — Figs. S1 to S8 Tables S1 to S4 [file research.0632.f1.zip › Fig. S3.tif]

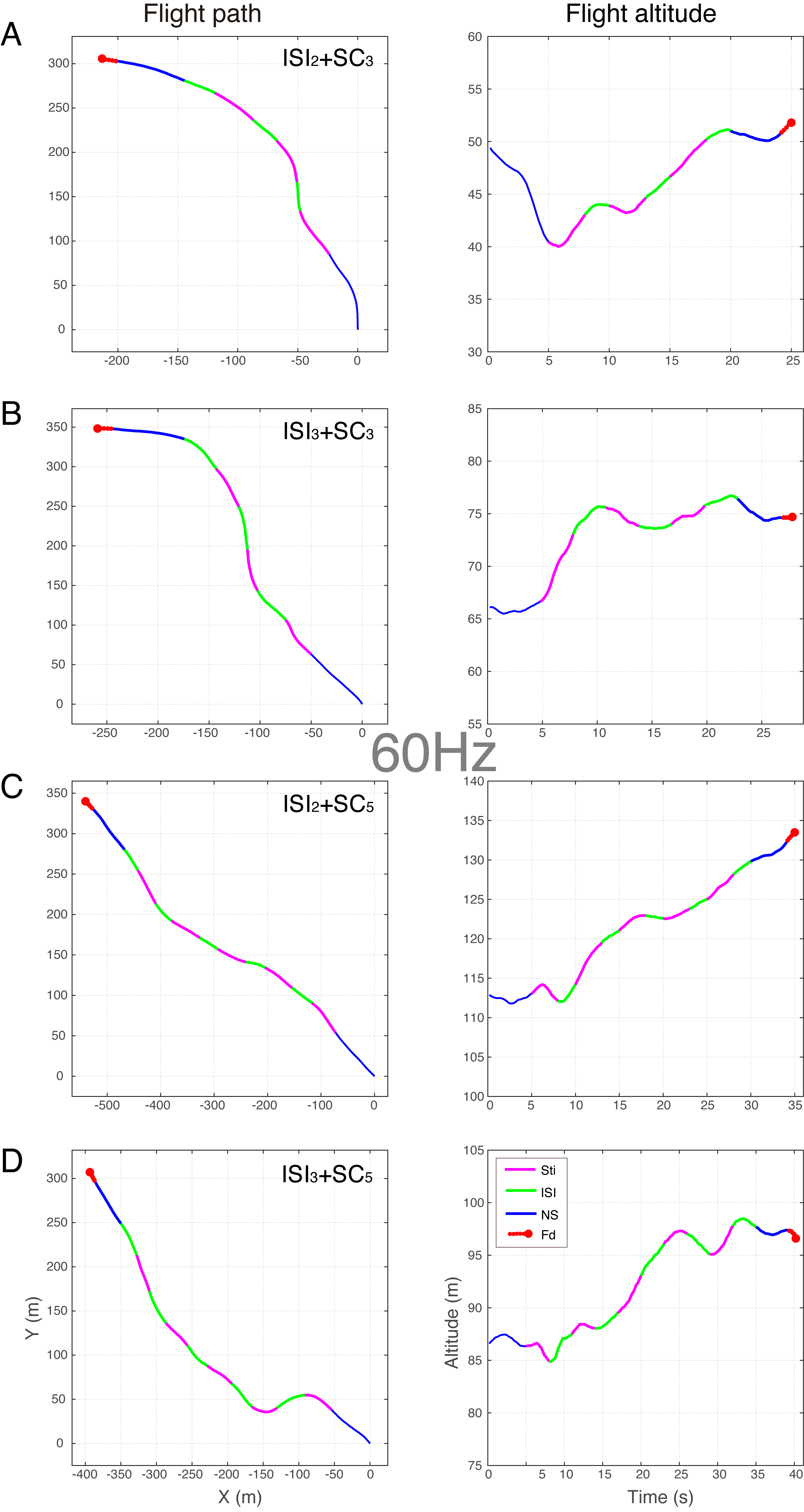

Supplement: Supplementary 1 — Figs. S1 to S8 Tables S1 to S4 [file research.0632.f1.zip › Fig. S4.tif]

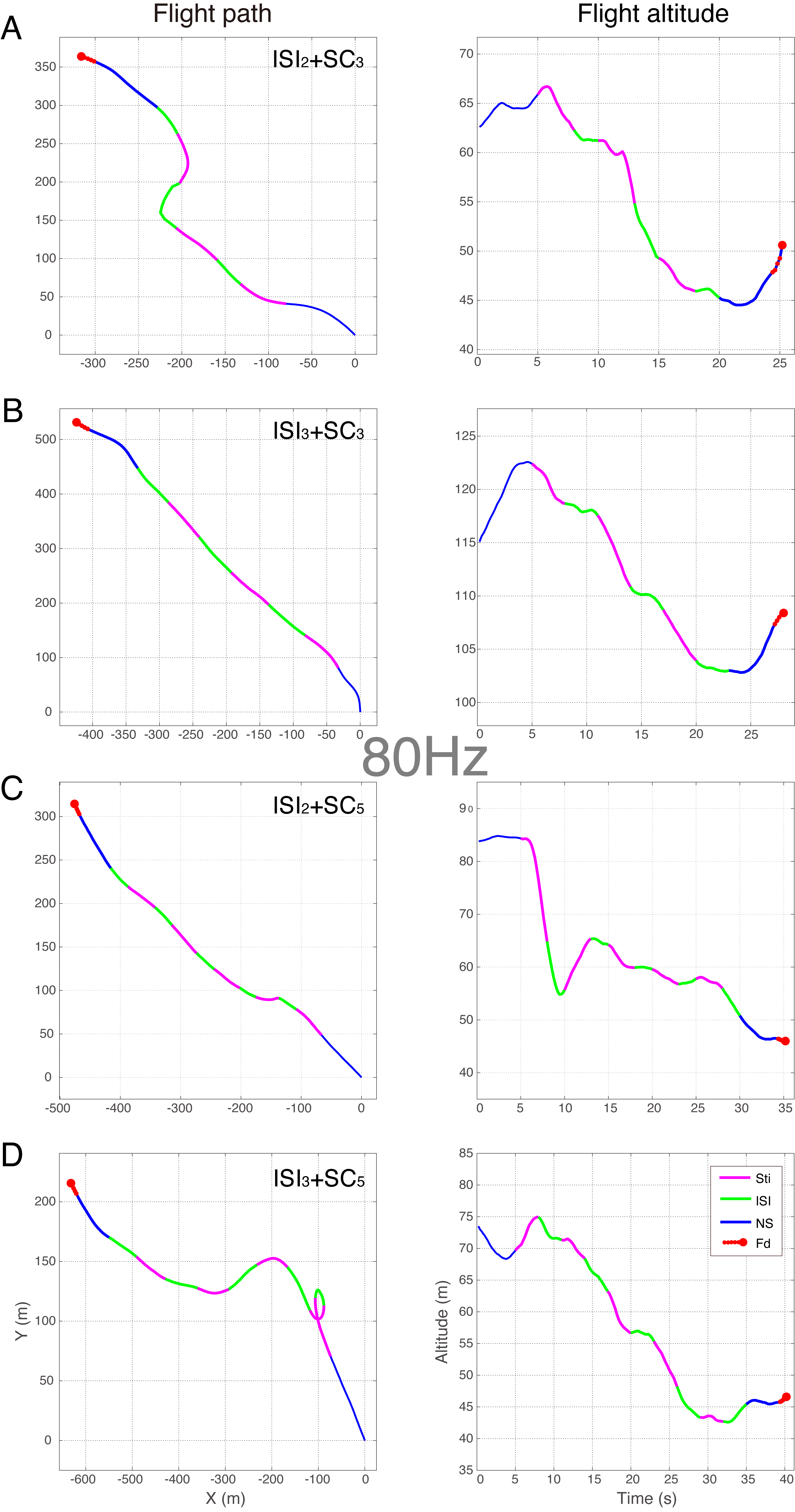

Supplement: Supplementary 1 — Figs. S1 to S8 Tables S1 to S4 [file research.0632.f1.zip › Fig. S5.tif]

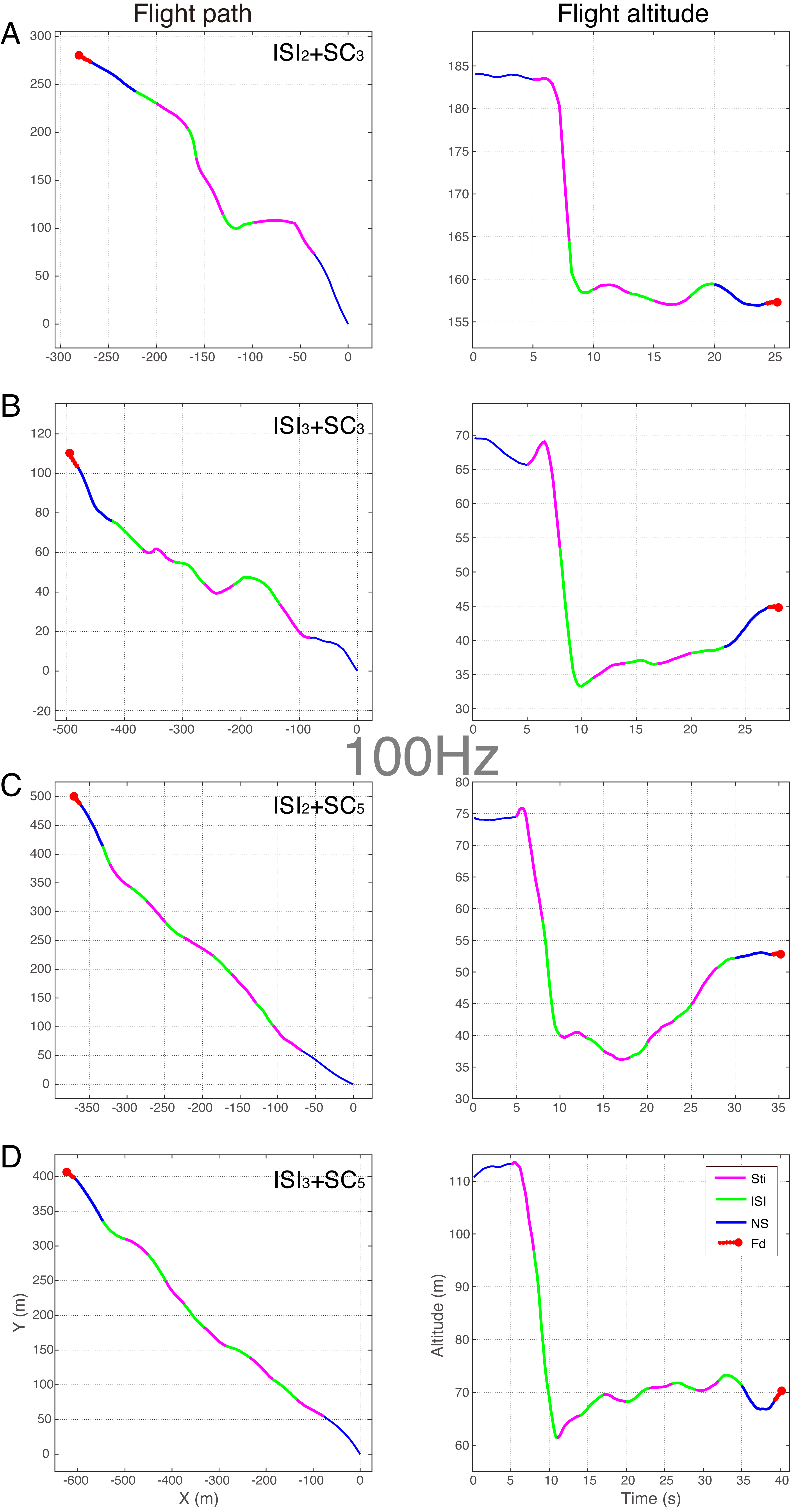

Supplement: Supplementary 1 — Figs. S1 to S8 Tables S1 to S4 [file research.0632.f1.zip › Fig. S6.tif]

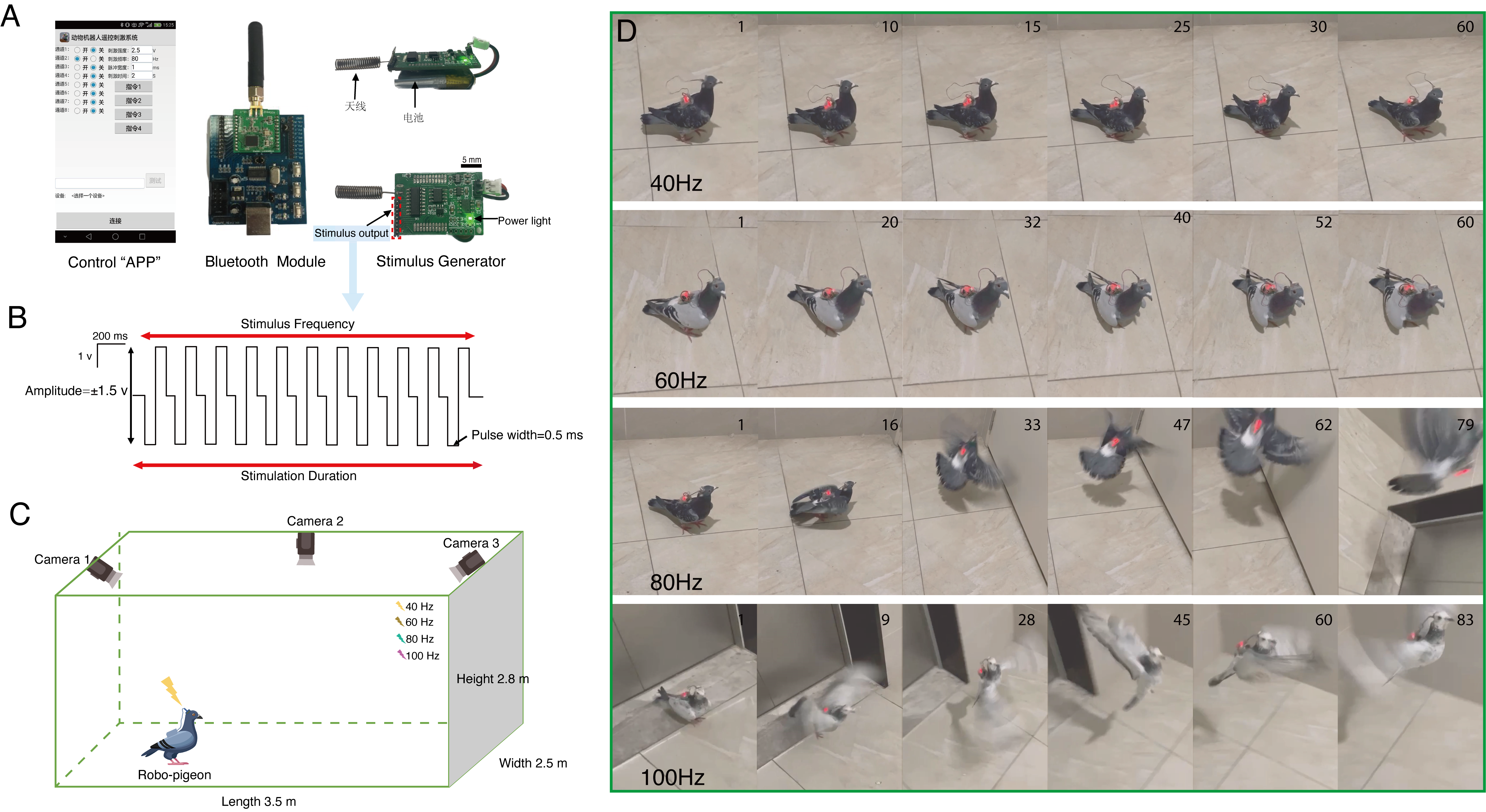

Supplement: Supplementary 1 — Figs. S1 to S8 Tables S1 to S4 [file research.0632.f1.zip › Fig. S7.tif]

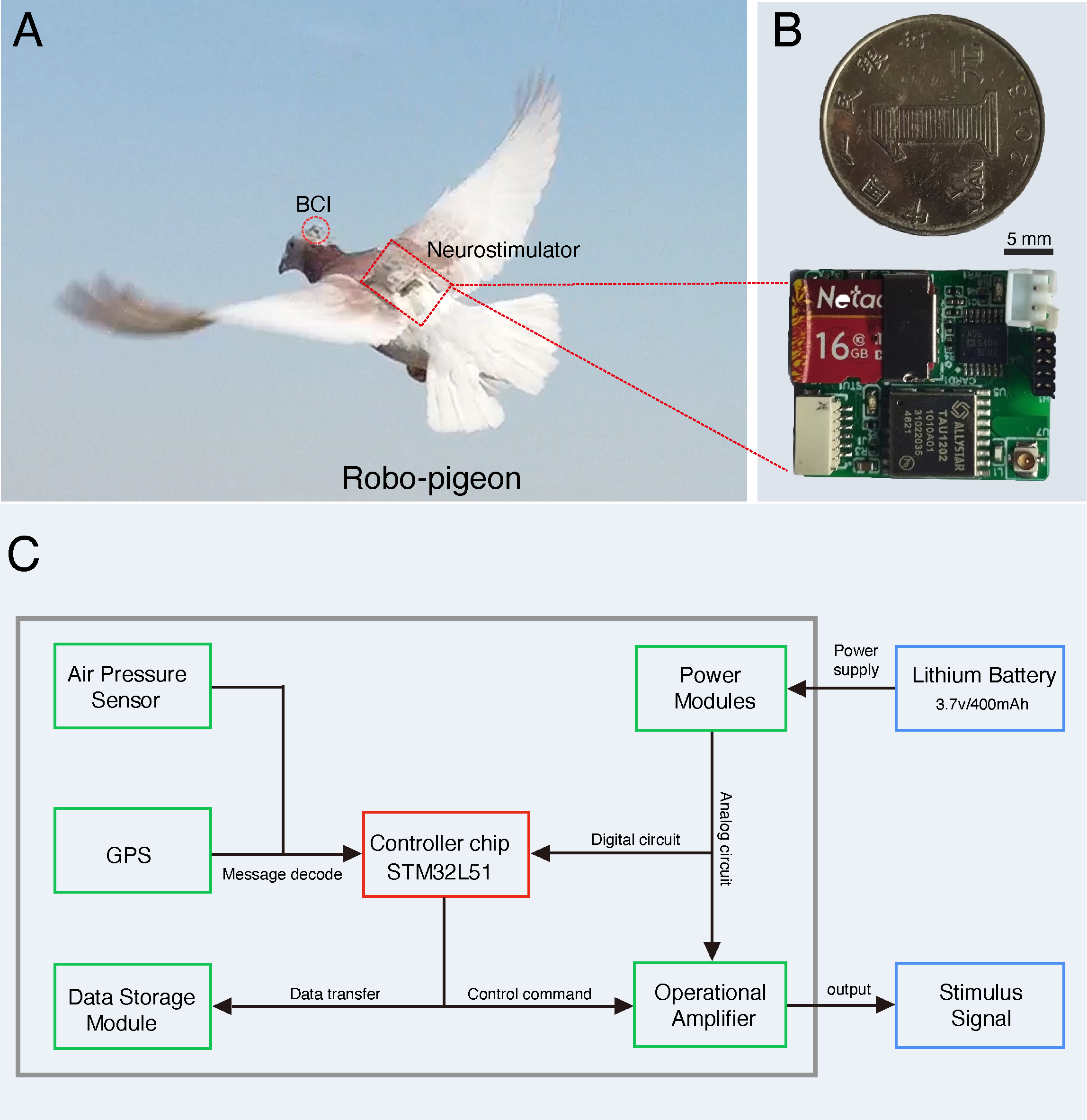

Supplement: Supplementary 1 — Figs. S1 to S8 Tables S1 to S4 [file research.0632.f1.zip › Fig. S8.tif]
